# Discovery of Novel Hybrid-Type Strigolactone Mimics Derived from Cinnamic Amide

**DOI:** 10.3390/ijms24129967

**Published:** 2023-06-09

**Authors:** Chunying Wang, Bingbo Guo, Zhaokai Yang, Lin Du, Chunxin Yu, Yuyi Zhou, Hanqing Zhao, Ye Wang, Liusheng Duan

**Affiliations:** 1 State Key Laboratory of Plant Physiology and Biochemistry, Engineering Research Center of Plant Growth Regulator, Ministry of Education & College of Agronomy and Biotechnology, China Agricultural University, Beijing 100193, China; chunying_wang@cau.edu.cn (C.W.);; 2 Innovation Center of Pesticide Research, Department of Applied Chemistry, College of Science, China Agricultural University, Beijing 100193, China; 3 College of Plant Science and Technology, Beijing University of Agriculture, Beijing 102206, China

**Keywords:** strigolactones, cinnamic amide, seed germination, root growth, lateral root formation, root hair elongation, molecular docking

## Abstract

Strigolactones (SLs) are a class of plant hormones and rhizosphere communication signals of great interest. They perform diverse biological functions including the stimulation of parasitic seed germination and phytohormonal activity. However, their practical use is limited by their low abundance and complex structure, which requires simpler SL analogues and mimics with maintained biological function. Here, new, hybrid-type SL mimics were designed, derived from Cinnamic amide, a new potential plant growth regulator with good germination and rooting-promoting activities. Bioassay results indicated that compound **6** not only displayed good germination activity against the parasitic weed *O. aegyptiaca* with an EC_50_ value of 2.36 × 10^−8^ M, but also exhibited significant inhibitory activity against *Arabidopsis* root growth and lateral root formation, as well as promoting root hair elongation, similar to the action of GR24. Further morphological experiments on *Arabidopsis max2-1* mutants revealed that **6** possessed SL-like physiological functions. Furthermore, molecular docking studies indicated that the binding mode of **6** was similar to that of GR24 in the active site of OsD14. This work provides valuable clues for the discovery of novel SL mimics.

## 1. Introduction

**Figure 1:**
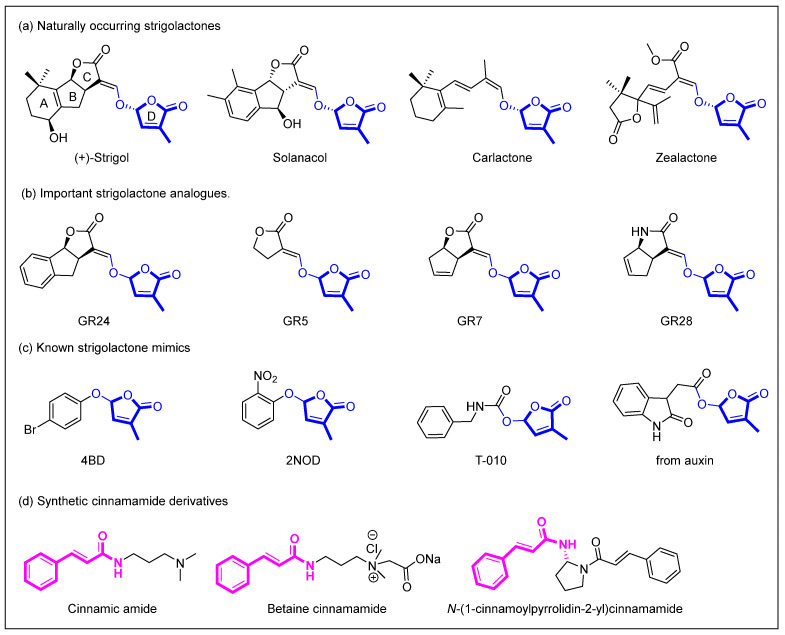
Chemical structures of natural SLs (**a**), some synthetic strigolactone analogues (**b**), artificial SL mimics (**c**), and some cinnamamide derivatives (**d**).

Strigolactones (SLs) are a new class of phytohormones with extensive biological activities that have attracted great attention in plant biology [1,2]. SLs were originally recognized as root-derived exogenous signals stimulating the germination of root parasitic weeds [3]. Additionally, recent studies have revealed that SLs are involved in the regulation of the root architecture (lateral root density, root hair elongation, primary root length) of model plants (*Arabidopsis*, *Oryza*, *Pisum*, etc.) [4,5,6] and the inhibition of bud outgrowth and shoot branching [2]. To date, more than 30 natural SLs have been isolated from host crops such as sorghum, maize, rice, and tobacco, and different monocrotalide mixtures can be generally secreted from different plant species [7,8]. SLs constitute a group of carotenoid derivatives containing a butenolide ring (D-ring) connected to a variable second structural via an enol ether bridge. On the basis of structure, SLs have been divided into two categories. Canonical SLs contain a tricyclic scaffold (ABC ring), such as strigol and solanacol (Figure 1a). Non-canonical SLs, which do not have an ABC-lactone for instance carlactone and zealactone (Figure 1a), are simper than canonical SLs [9,10].

Nevertheless, the availability of natural SLs is limited to a large extent by the extremely minute amounts in root exudates. Furthermore, the total synthesis of natural SLs is challenging, as their structures are rather complex [4,11]. As a consequence, the development of SL analogs with simplified structures and essential bioactivities has become a hot research topic in recent years. A series of SL analogs, including GR24, GR28, GR5, GR7 (Figure 1b), have been reported [12]. The most typical example of these analogs is GR24, which is widely used as a bioassay standard. Regrettably, the complicated synthesis steps, high cost, and low stability of GR24 has limited its practical use [1,13]. In addition, some studies have reported a novel class of SL mimics that exert similar biological functions to SL analogs but lack the ABC scaffold and only have a substituent on C-5 of the lactone ring (D-ring), such as 4-bromodebranone (4BD) [14], 2-nitrodebranone (2NOD) [15] or T-010 [16], and an SL analogue derived from auxin (Figure 1c) [17]. To the best of our knowledge, a family of α/β-hydrolases have been proposed to be SL receptor proteins [18]. During the signaling process, SLs are first perceived by the α/β-hydrolase D14 and subsequently undergo a D14-triggering open-to-closed transition state pathway to release the active lactone D-ring [19,20]. The mechanism of action of these SL mimics can be explained as the mimics interacting with the D14 receptor, resulting in the detachment of the D-OH ring, which leads to a series of cascade reactions and triggers the signal transmission [19,20,21]. Moreover, these mimics, which have greater stability, easy synthesis, and a simple structure, are more conducive to the practical application. Therefore, the D-ring should be retained as an important core fragment in the design of SL mimics.

Despite the fact that the synthesis and bioactivity of SL mimics have been reported, the knowledge concerning them is still at an early stage. A variety of new hybrid-type SL mimics have been designed and synthesized based on the structures of plant growth regulators. Pereira et al. [22] reported a range of hybrid SL mimics from the phytohormone gibberellic acid (GA3), which had good biological activity against parasitic weed seed germination; Zwanenburg et al. [17,23] subsequently developed new hybrid SL mimics derived from auxin, which were shown to be effective germination stimulants. It is noteworthy that a wide range of studies have taken place on SL mimics derived from plant growth regulators as exogenous signals for rhizosphere organisms, while their activities in terms of phytohormones have rarely been reported.

**Scheme 1:**
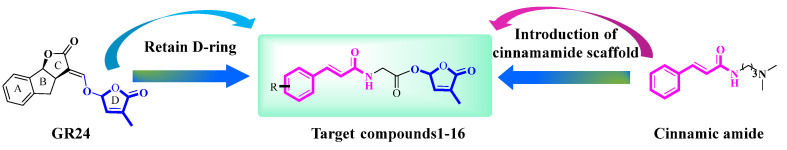
Design strategy of the target compounds.

Cinnamamides are an important class of natural active molecules, and the cinnamamide scaffold is a vital structural unit for maintaining their biological activities [24]. Cinnamamide derivatives are involved in a wide spectrum of biological activities such as antitumor [25], antioxidant [26], antiviral [27], seed germination, and rooting-promoting activities [28]. It has been reported that Cinnamic amide and its derivative Betaine cinnamamide (Figure 1d) were considered as novel plant growth regulators, which can preferably promote wheat seed germination, accelerate root and shoot growth, and improve wheat quality [29]. Subsequently, a novel plant growth regulator derived from the edible mushroom *Pholiota lubrica*, *N*-(1-cinnamoylpyrrolidin-2-yl) cinnamamide, was reported to inhibit the growth of hypocotyl and root of lettuce [30]. These results suggested that cinnamamide scaffolds can be used as a significant template for designing new plant growth regulators. Herein, 16 novel hybrid-type SL mimics based on the structure of Cinnamic amide combined with the D-ring were designed and synthesized in the present work (Scheme 1). These target compounds had an amide motif as a linker, as with T-010 (Figure 1c). However, they have a unique cinnamamide scaffold compared with it. We also evaluated their seed germination activity against the parasitic weed and phytohormonal activity of *Arabidopsis*. Furthermore, their mechanisms to target OsD14 were illustrated using the molecular docking method.

## 2. Results and Discussion

### 2.1. Synthesis of Target Compounds ***1**–**16***

**Scheme 2:**
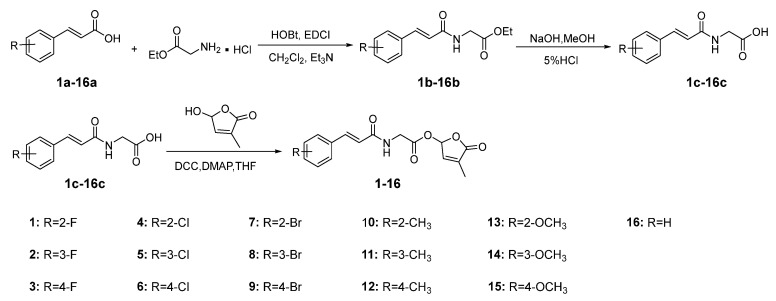
Synthetic route of target compounds **1**–**16**.

A series of new, hybrid-type SL mimics were synthesized from simple and cheap commercially available materials. The synthetic route adopted for the synthesis of the target compounds is shown in Scheme 2. The intermediates **1b**–**16b** were prepared via a condensation reaction using 1-hydroxybenzotrizole (HOBt) and 1-(3-Dimethylaminopropyl)-3-ethylcarbodiimide hydrochloride (EDCI) in moderate yields. Subsequently, these intermediates were hydrolyzed in aqueous sodium hydroxide, and the pH was adjusted to pH 3 using 5% hydrochloric acid to obtain intermediates **1c**–**16c** in good yields [31]. Finally, the target compounds **1**–**16** were acquired through the esterification of 5-hydroxy-3-methyl-2(5H)-furanone with the substituted cinnamoylglycine, using dicyclohexylcarbodiimide (DCC) as a condensation agent and 4-dimethylaminopyridine (DMAP) as a catalyst. All these syntheses conform to the criteria of operational simplicity: starting with inexpensive, commercially available compounds, only three synthetic steps are required. The structures of all the newly prepared hybrid-type SL mimics were confirmed through the analyses of their melting points, ^1^H NMR, ^13^C NMR, and HRMS, and physical data are presented in Section 3.2.

### 2.2. The Biological Activity of Target Compounds

#### 2.2.1. The Effect on Parasitic Weed Seeds

The synthetic SL mimics derived from Cinnamic amide were first bioassayed for their ability to stimulate seed germination of the parasitic plant O. aegyptiaca. The germination rate was acquired with the assistance of a microscope [32]. The germination rates of compounds **1**–**16** and positive control GR24 (racemic mixture) and their half-maximal effective concentration (EC_50_), which was used to evaluate for their effect on parasitic weed seeds, are shown in Table 1.

**Figure 2:**
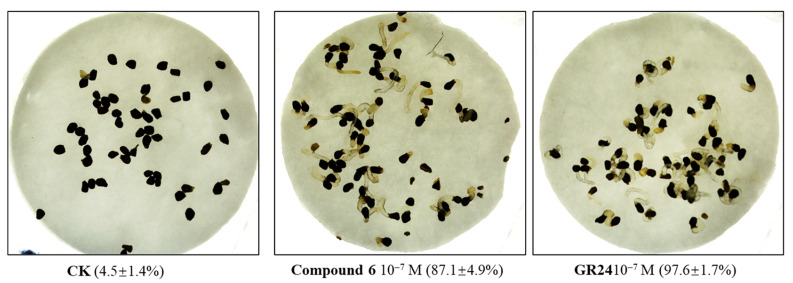
Seed germination activity of compound **6** and GR24 against *O. aegyptiaca*.

From the results, it was clear that all of the target compounds could promote seed germination in *O. aegyptiaca* to varying degrees at 10^−5^, 10^−6^, 10^−7^ M. Among the 16 target compounds, most of them showed moderate-to-high germination activity at a concentration of 10^−5^ M. In particular, compounds **5** (3-Cl), **6** (4-Cl), **7** (2-Br), and **8** (3-Br) exhibited germination rates of more than 80%, which were similar to that of GR24. Moreover, compounds **5** and **6** continued to achieve germination rates of more than 80% at a lower dosage of 10^−6^ M. These compounds showed an EC_50_ value ranging from 2.36 to 8.02 × 10^−8^ M (compounds **5**, **6**, **7,** and **8**, Table 1). Remarkably, even at a concentration of 10^−7^ M, compound **6** reached a germination rate of 87.1% and an EC_50_ value of 2.36 × 10^−8^ M, exhibiting the highest stimulatory potentiality among these target compounds. Although its overall activity was about one order of magnitude weaker than that of GR24 (EC_50_, 3.09 × 10^−9^ M), these discoveries are still significant. An overview of the germinated seeds treated with compound **6** and GR24 at 10^−7^ M are shown in Figure 2.

Furthermore, an analysis of the preliminary structure–activity relationships (SARs) of the compounds revealed that the type and position of the substituents on the benzene ring had a significant impact on the activity. The germination activities of compounds with an electron-withdrawing substituent (F, Cl) on the benzene ring were more active than those without a substituent (H): for instance, **6** (4-Cl) > **3** (4-F) > **16** (H). Compounds with an electron-withdrawing substituent at 4-position (F, Cl) seemed to possess better activity than those with an electron-donating substituent (CH_3_, OCH_3_). For example, **6** (4-Cl) > **3** (4-F) > **15** (4-OCH_3_) > **12** (4-CH_3_). Meanwhile, when the substituents on the benzene ring were fluorine and chlorine, compounds in the para position of the benzene ring exhibited much higher activities than those in other positions: for example, **3** (4-F) > **2** (3-F) > **1** (2-F), **6**(4-Cl) > **5** (3-Cl) > **4** (2-Cl). Altogether, these results indicated that the introduction of an electron-withdrawing substituent on the para position of the benzene ring was beneficial for the germination activity. In particular, chlorine substituent was shown to have a positive effect on the seed germination activity.

#### 2.2.2. Evaluation of Effect on *Arabidopsis* Roots

As phytohormones, strigolactones can regulate the shoot and root architecture of model plants (*Arabidopsis*, rice, pea, etc.). It has been reported that strigolactones exert potent effects on the primary root elongation and lateral root generation of *Arabidopsis* [33]. In this study, *Arabidopsis* was tested with all of the synthesized SL mimics at three concentrations (1, 50, and 100 μM) in order to explore the phytohormonal activity of the synthetic mimics on *Arabidopsis* roots. The inhibition rates and the IC_50_ (half-maximal inhibitory concentration) values of compounds **1***–***16** on the length of *Arabidopsis* (wild-type Columbia-0, Col-0) primary roots are listed in Table 2.

In total, the results clearly demonstrated that most of the tested compounds exhibited definite inhibitory activities against primary root elongation at 50 and 100 μM, and the inhibition effect of compounds depended on their applied concentration, with higher concentrations resulting in stronger inhibitory activity. Of particular note, compound **6**, which showed the best seed germination activity among these compounds, also presented the highest inhibitory potential of primary root elongation with an IC_50_ value of 25.21 μM.

Specifically, at a concentration of 100 μM, all compounds except **5**, **7**, **11**, **14**, and **16** displayed inhibition rates of more than 70% on primary root elongation. In particular, compound **6** (91.3%) significantly inhibited the primary root length of *Arabidopsis* to an extent similar to the effect exerted by GR24 (96.6%). The inhibitory activities of some compounds at higher concentrations may be attributed to their toxicity at nonphysiological concentrations, which were consistent with that of GR24 reported in previous study [34,35]. At 50 μM, as much as over 50% inhibition of primary root elongation was obtained for most of the tested compounds. For example, compound **6** achieved an inhibition rate of up to 83.2%. However, when the concentration was as low as 1 μM, the inhibitory effect of most of the compounds on *Arabidopsis* primary root growth was diminished or switched to a promoting effect. For example, compound **6** (2.7%) and GR24 (3.0%) exerted extremely weak inhibitory activities at 1 μM, which can be ignored, while compound **1** stimulated a primary root elongation of 17.4%. This phenomenon is consistent with the results previously reported in the literature [34].

At the same time, the different activities of these compounds unraveled that the position and type of substituents on the benzene ring also had a significant influence on the growth of *Arabidopsis* primary roots. When the substituent R was of an electron-withdrawing group (fluorine, chlorine) or an electron-donating group (methyl, methoxy), compounds with the substituent in the para position showed higher activity than those in the other position. For instance, **12** (4-CH_3_) > **10** (2-CH_3_) > **11** (3-CH_3_). The position of the modification is conclusive. When the position of the substituent on the benzene ring was para, the chlorine substitution had the best inhibitory effect, which was remarkably higher than that of other types of substituents: for example, **6** (4-Cl) > **12** (4-CH_3_) > **3** (4-F) > **15** (4-OCH_3_) > **9** (4-Br).

**Figure 3:**
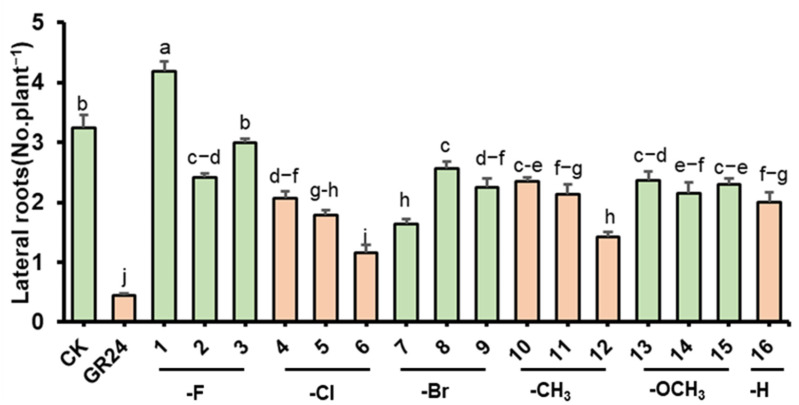
The effect of Compounds **1**–**16** on wild-type *Arabidopsis* lateral root density. Number of lateral roots per plant after compound application. Eight-day-old seedlings (at least 30) treated with compounds were photographed digitally, and then measurement was carried out using ImageJ software (https://imagej.nih.gov/ij/index.html, accessed on 11 December 2022). A one-way ANOVA followed by a Duncan’s test was used for significant differences at *p* < 0.05, marked with lowercase letters. Different lowercase letters indicate significant differences.

Considering the reported results that GR24 can reduce the number of lateral roots at 10 μM [14], we also evaluated the effect of these compounds on wild-type *Arabidopsis* lateral root density at the same concentration, and the experimental results are shown in Figure 3. Compounds **5**, **6**, **7**, **11**, **12**, and **16** and GR24 significantly inhibited the lateral root formation of *Arabidopsis* at 10 μM when compared to the control. The most efficient compound was **6**, which displayed a slightly weaker activity than that of GR24. On the contrary, we discovered that compound **1** induced lateral root generation. In general, this observation was relevant to the previously published results reporting that other SL mimics (4-bromodebranone, triazolide strigolactone mimics) [14,21], which have an acyclic unsaturated system connected to a D-ring, had an impact on the lateral root formation of *Arabidopsis*.

#### 2.2.3. Effect of Compound **6** on *Arabidopsis* Mutant Growth

**Figure 4:**
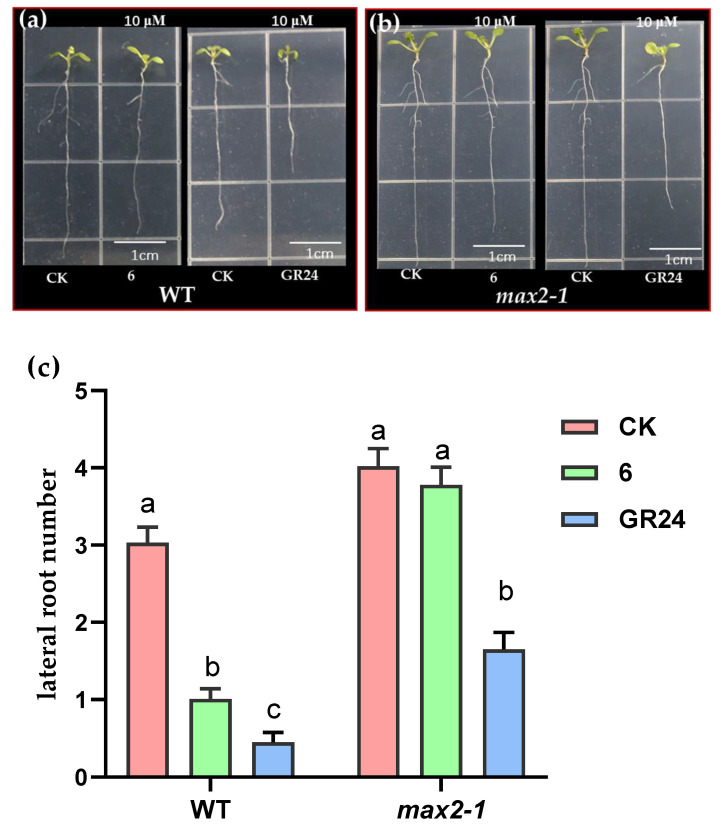
The effect of **6** on *Arabidopsis* lateral root density. (**a**) Eight-day-old wild-type (WT) *Arabidopsis* with or without 10 µM chemical treatment. Scale bar indicates 1 cm. (**b**) Eight-day-old *max2-1* mutant with or without 10 µM chemical treatment. Scale bar indicates 1 cm. (**c**) Number of lateral roots in wild-type (WT) and *max2-1* mutant *Arabidopsis* with or without 10 µM chemical treatment. The data are means ± standard deviation of forty samples. A one-way analysis of variance (ANOVA) was performed, followed by Duncan’s test (Different letters indicate values that are significantly different (*p* < 0.05)).

**Figure 5:**
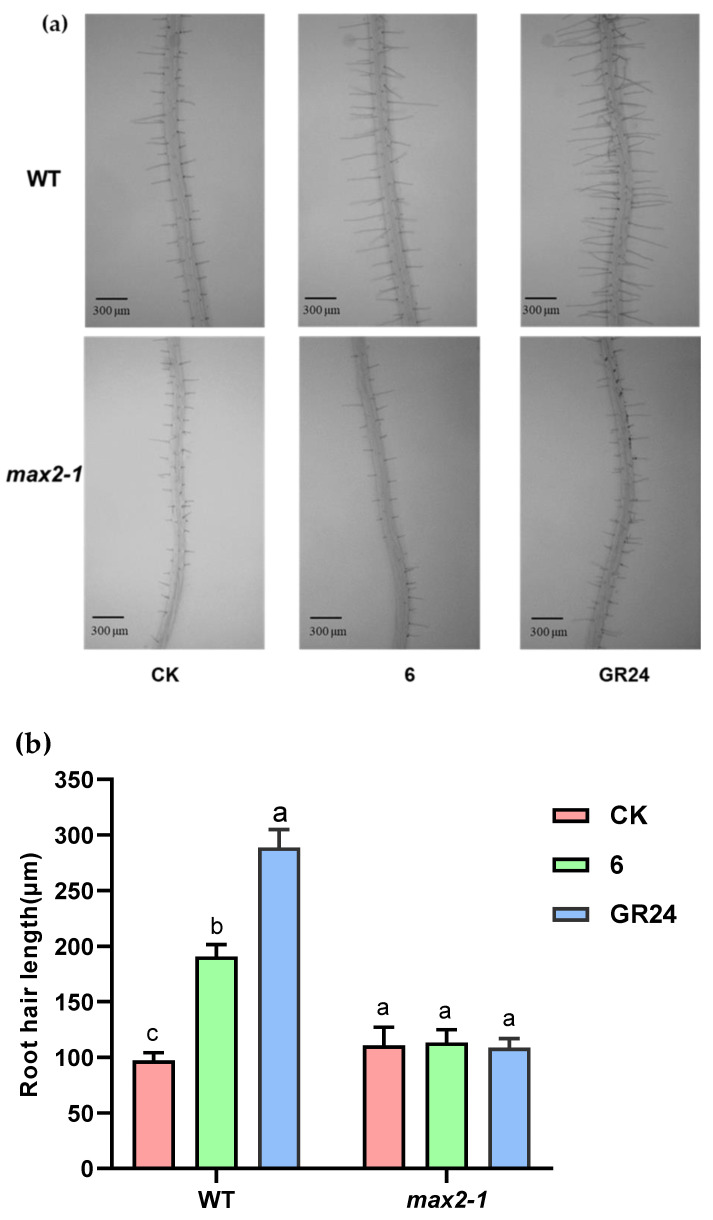
The effect of **6** on *Arabidopsis* root hair elongation. (**a**) Root hair of wild type (WT) and *max2-1* with or without the 10 μM chemical treatment. Scale bars indicate 300 μm. (**b**) Root hair length of eight-day-old *Arabidopsis* with or without 10 μM chemical treatment. The data are means ± standard deviation (n = 15 roots; 10 root hairs per root were measured). A one-way analysis of variance (ANOVA) was performed, followed by Duncan’s test (Different letters indicate values that are significantly different (*p* < 0.05)).

In *Arabidopsis*, SLs are perceived by the α/β-hydrolase D14, which interacts with the F-box protein MAX2, and subsequently hydrolyzed via a D14-triggering state pathway [18,19]. Several studies have demonstrated that MAX2 functions in the root-specific SL signaling pathway [14]. In these previous studies, SL did not induce the lateral roots in the SL-signaling mutant *max2*. However, SL significantly increased the root hair length in the wild type (WT) but not in the *max2* mutant. Due to the high sensitivity of SLs to *Arabidopsis* roots, we believe that this biological system should be suitable for assessing the activity of SL mimics. Therefore, we examined the effects of compound **6** on *Arabidopsis* lateral root formation (Figure 4) and root hair elongation (Figure 5). The experimental results are presented below.

For lateral root formation, in the WT, compound **6** obviously reduced the lateral root number at a concentration of 10 μM, but the effect of **6** was weaker than that of GR24 (Figure 4a,c). In the *max2-1* mutant, treatment with GR24 affected the lateral root growth at a concentration of 10 μM, although **6** displayed no effect (Figure 4b,c). A possible reason for this effect may be an adverse effect of GR24 or another signaling pathway that can recognize high concentrations of GR24, other than that which pass through MAX2. These results demonstrated that **6** inhibited *Arabidopsis* lateral root formation in a MAX2-dependent manner, which was consistent with the previously reported 4BD [14]. 

For the root hair, in the WT, compound **6** significantly promoted the elongation of the root hairs more so than untreated samples at a concentration of 10 μM, but the effect of **6** was weaker than that of GR24 (Figure 5a,b). In the *max2-1* mutant, neither **6** nor GR24 promoted the growth of root hairs (Figure 5a,b). These results suggested that **6** promoted root hair elongation in a MAX2-dependent manner. However, the direct effect of the target compounds on SL signaling needs further investigation. Therefore, the SL-like activity of compound 6 at the transcript level in *Arabidopsis* roots revealed by gene expression experiments warrants further study.

### 2.3. Molecular Docking of Ligands to OsD14

In order to explore the detailed molecular mechanisms of cinnamic amide SL mimics with an SL receptor, compounds **6** and **7** were chosen as typical ligands to rationalize the more efficient impact on parasitic weed seeds and *Arabidopsis* root, while **4**–**6** were selected as representative compounds to reveal the relationship between the different positions of the same substituent and the binding affinities. OsD14 (PDB: 5DJ5) was chosen as the protein receptor for molecular docking due to the lack of a crystal structure for the SL receptor in *O. aegyptiaca* and *Arabidopsis* [15,35].

**Figure 6:**
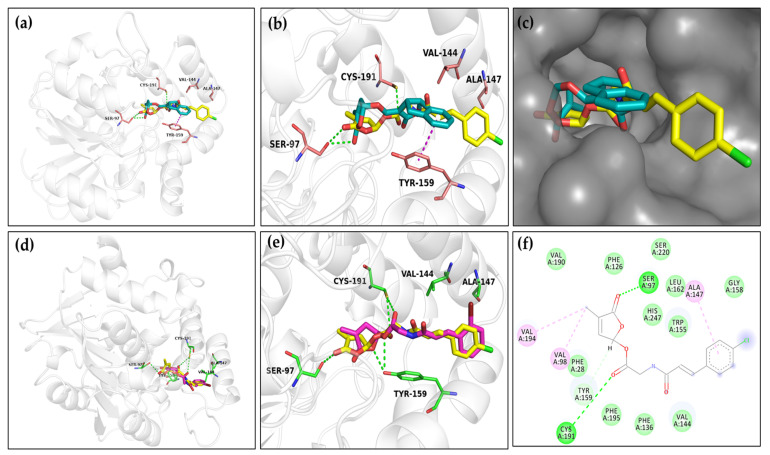
Docking modes of compounds **6** and **7** with OsD14. (**a**) Pocket location of OsD14 when compound **6** and GR24 combined on it. (**b**) Close-up view of compound **6** as yellow sticks bound in the catalytic site of OsD14. (**c**) View from the top of the catalytic pocket, with compound **6** shown as yellow sticks. (**d**) Pocket location of OsD14 when compounds **6** and **7** combined on it. (**e**) Comparison of the binding postures of compounds **7** (purple sticks) and **6** (yellow sticks) bound in the catalytic site of OsD14. (**f**) A 2D view of compound **6** in the catalytic site of OsD14. Note: The cyan sticks shown in (**a**–**c**) are GR24, which is the original ligand of OsD14.

From the docking results, we conclude that both compound **6** and GR24 were oriented into the pocket, and their D-rings in the active site showed the same orientation and exhibited similar binding patterns (Figure 6a,c). As illustrated in Figure 6b, the carbonyl groups on the butenolide moieties of compound **6** and GR24 all reached toward Ser 97 to form hydrogen bonds, which was essential for the hydrolysis of the mimics. Furthermore, the carbonyl group on the chain near the D-ring moiety was able to form a hydrogen bond with Cys 191, whereas the aromatic ring at the pocket entrance formed a pi-Alkyl interaction with Ala 147 (Figure 6f). The above interactions may be responsible for the high activity of compound **6**. However, compared to GR24, the phenyl ring of compound **6** extended to the outside of the pocket, which may account for the fact that the activity of **6** were slightly lower than that of GR24. Therefore, it is beneficial to improve the activity of the mimics by appropriately shortening the length of the bridge chain in subsequent study.

In addition, we also compared molecular docking on compounds **6** and **7** with OsD14, and the different conformations are presented in Figure 6d. Compound **7** could form hydrogen bonding interactions with Cys 191 and Tyr 159, and its D-ring orientation was consistent with that of compound **6** (Figure 6e). However, the carbonyl group in the D-ring of compound **7** did not form a hydrogen bond with Ser 97, which probably explains the slightly weaker activity of compound **7** in comparison with compound **6**.

**Figure 7:**
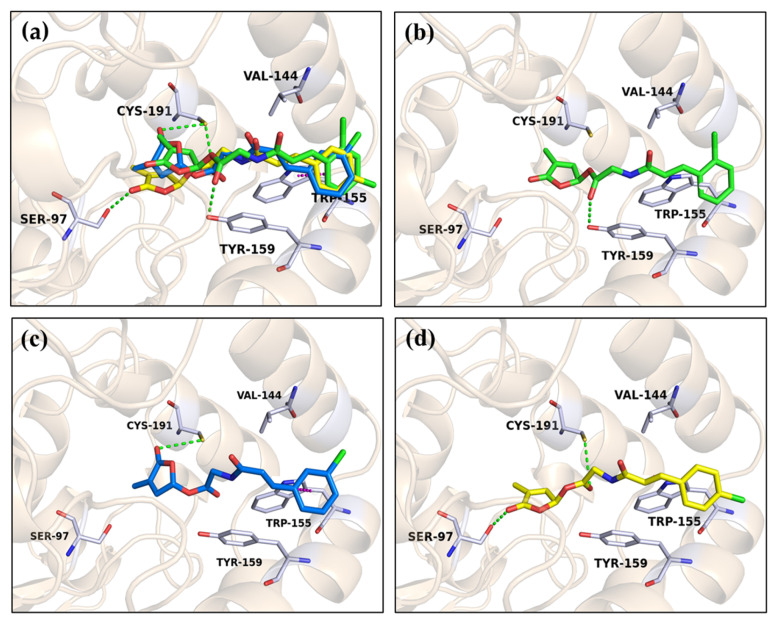
(**a**) Superimposed conformations of the molecular docking ligands. (**b**) The docking conformation of **4** (ortho, green). (**c**) The docking conformation of **5** (meta, blue). (**d**) The docking conformation of **6** (para, yellow).

Furthermore, the different positions of the same substituent could significantly affect the binding interaction. As shown in Figure 7a, similar to compound **6** (4-Cl), the D-rings of compounds **4** (2-Cl) and **5** (3-Cl) were located near the center of the active site pocket of OsD14. A hydrogen bond interaction existed between Tyr 159 in OsD14 and the carbonyl group on the chain near the D-ring of compound **4** (Figure 7b), and a hydrogen bond interaction existed between Cys 191 in OsD14 and the carbonyl group in the D-ring of compound **5** (Figure 7c). Notably, neither compound **4** nor **5** formed a hydrogen bond with Ser 97, while compound **6** could form the key hydrogen bond interaction with Ser 97 (Figure 7d). Thus, compound **6** presented more active bioactivity than the other two.

### 2.4. Hydrolytic Stability

To obtain a complete picture of these compounds, stability tests were performed on compound **6** and GR24 in aqueous solutions at different pH values (from 5 to 8). As illustrated in Figure S1, compound **6** was found to be more stable in acidic conditions (pH 5) than neutral (pH 7) and basic conditions (pH 8), similarly to GR24. For example, about 30% of compound **6** was hydrolyzed in acidic (pH 5) conditions after 1 day, whereas complete hydrolysis within 1 day was observed in neutral (pH 7) and basic conditions (pH 8). By comparison with the compound **6**, GR24 was approximately 30% and 75% hydrolyzed after 7 days in acidic (pH 5) and neutral (pH 7) conditions, respectively, while it was completely hydrolyzed within 1 day in basic conditions (pH 8). These results suggested that compound 6 was less stable than GR24, which may be the reason why the activity of **6** was weaker than that of GR24.

## 3. Materials and Methods

### 3.1. General Information

All solvents and reagents (of analytical grade) were purchased from the HEOWNS Corporation (Tianjin, China) and used without further purification. Silica gel (200–300 mesh, Puke Corporation, Qingdao, China) was used for column chromatographic purification. The ^1^H NMR spectra and ^13^C NMR spectra were obtained on an AVANCE NEO 500 M spectrometer (Bruker, Bremen, Germany) using dimethyl sulfoxide-*d*_6_ (DMSO-*d*_6_) as a solution and tetramethylsilane (TMS) as an internal standard. High-resolution mass spectrometry (HRMS) data regarding the target compounds were obtained using a 7.0T FTICR-MS instrument (Varian, Palo Alto, CA, USA). The melting points of all of the compounds were determined using a Stuart SMP3 melting point apparatus and were uncorrected. The *Arabidopsis* growth data were obtained by using ImageJ software (https://imagej.nih.gov/ij/index.html, accessed on 11 December 2022).

The seeds of *O. aegyptiaca* were kindly provided by Wei He (Research Institute of Plant Protection, Xinjiang Academy of Agricultural Sciences, Urumqi, China). *Arabidopsis* (A. thaliana ecotype Columbia-0, Col-0) were provided by the State Key Laboratory of Plant Physiology and Biochemistry, College of Agronomy and Biotechnology, China Agricultural University, Beijing, China. *Arabidopsis thaliana* more axillary shoot mutants *max2-1* were kindly provided by Prof. Shuhua Yang (State Key Laboratory of Plant Physiology and Biochemistry, College of Biological Sciences, China Agricultural University, Beijing, China).

### 3.2. Synthesis of Target Compounds ***1**–**16***

To a solution of HOBt (24.72 mmol) and EDCI (24.82 mmol) in dichloromethane (40 mL) at room temperature, the corresponding cinnamic acid was added (16.48 mmol), and the mixture was stirred for 1.5 h. Then, a solution of glycine ethyl ester hydrochloride (24.72 mmol) in dichloromethane (50 mL) was added dropwise, and the pH of the solution was adjusted to pH 7 with triethylamine. The resulting mixture was then stirred overnight at room temperature and monitored via TLC. After the completion of the reaction, the solution was extracted with dichloromethane, and the organic phase was sequentially washed with saturated NaCl aqueous solution, dried with anhydrous sodium sulfate, and filtered, and the solvent was then evaporated under reduced pressure. The residue was purified via column chromatography (eluent: petroleum ether/ethyl acetate = 3:1, *v*/*v*) to obtain ethyl cinnamoylglycinate intermediates **1b**–**16b**.

To a solution of ethyl cinnamoylglycinate (7.49 mmol) in 30 mL of methanol, a few drops of NaOH (6 mol/L) solution were added, and the mixture was stirred overnight at room temperature and monitored via TLC. After the completion of hydrolysis, the resulting mixture was concentrated. The residue was diluted with ice water (50 mL) and acidified to pH 3 with 5% dilute hydrochloric acid to obtain cinnamoylglycine intermediates **1c**–**16c**.

The cinnamoylglycine (4.18 mmol) was added to the solution of dicyclohexylcarbodiimide (DCC, 6.27 mmol) and 4-dimethylaminopyridine (DMAP, 6.27 mmol) in THF (30 mL) and stirred for 30 min. Then, 5-hydroxy-3-methyl-2-(5H)-furanone (5.02 mmol) diluted in THF (30 mL) was added to the mixture, and the mixture was stirred vigorously for 12 h at room temperature and monitored via TLC. After the complete consumption of the starting materials, the solution was filtered, and the filtrate was extracted with dichloromethane. Then, the organic layer was dried with anhydrous sodium sulfate, filtered, and evaporated under reduced pressure. The residue was finally purified via silica gel column chromatography to obtain the target Compounds **1**–**16**.

*4-methyl-5-oxo-2,5-dihydrofuran-2-yl(E)-(3-(2-fluorophenyl)acryloyl)glycinate* (**1**), white solid, m.p. 142–143 °C, 51% yield. ^1^H NMR (500 MHz, DMSO-*d*_6_) *δ* 8.78 (t, *J* = 5.9 Hz, 1H), 7.75–7.24 (m, 6H), 6.98–6.93 (m, 1H), 6.83 (d, *J* = 16.0 Hz, 1H), 4.16–4.05 (m, 2H), 1.89 (t, *J* = 1.4 Hz, 3H). ^13^C NMR (126 MHz, DMSO-*d*_6_) *δ* 171.64, 169.18, 165.82, 160.99 (d, *J* = 250.7 Hz), 144.37, 133.36, 132.55, 132.04 (d, *J* = 8.5 Hz), 129.73, 125.50, 124.41 (d, *J* = 5.9 Hz), 122.75 (d, *J* = 11.5 Hz), 116.58 (d, *J* = 21.6 Hz), 93.31, 41.24, 10.58. HRMS (ESI) *m*/*z* calcd for C_16_H_15_FNO_5_ [M + H]^+^: 320.0929; found, 320.0933.

*4-methyl-5-oxo-2,5-dihydrofuran-2-yl(E)-(3-(3-fluorophenyl)acryloyl)glycinate* (**2**), white solid, m.p. 179–180, 46% yield. ^1^H NMR (500 MHz, DMSO-*d*_6_) *δ* 8.66 (t, *J* = 5.9 Hz, 1H), 7.51–7.32 (m, 5H), 7.27–7.20 (m, 1H), 6.99–6.91 (m, 1H), 6.77 (d, *J* = 15.9 Hz, 1H), 4.17–4.04 (m, 2H), 1.89 (t, *J* = 1.4 Hz, 3H). ^13^C NMR (126 MHz, DMSO-*d*_6_) *δ* 171.65, 169.19, 165.77, 162.93 (d, *J* = 243.8 Hz), 144.38, 138.92 (d, *J* = 2.0 Hz), 137.74 (d, *J* = 7.9 Hz), 133.36, 131.39 (d, *J* = 8.7 Hz), 124.34 (d, *J* = 2.1 Hz), 123.18, 116.83 (d, *J* = 21.3 Hz), 114.55 (d, *J* = 21.6 Hz), 93.31, 41.24, 10.59. HRMS (ESI) *m*/*z* calcd for C_16_H_15_FNO_5_ [M + H]^+^: 320.0929; found, 320.0930.

*4-methyl-5-oxo-2,5-dihydrofuran-2-yl(E)-(3-(4-fluorophenyl)acryloyl)glycinate* (**3**), white solid, m.p. 161–162 °C, 53% yield. ^1^H NMR (500 MHz, DMSO-*d*_6_) *δ* 8.63 (t, *J* = 5.9 Hz, 1H), 7.70–7.64 (m, 2H), 7.48 (d, *J* = 15.8 Hz, 1H), 7.36–7.24 (m, 3H), 6.98–6.92 (m, 1H), 6.67 (d, *J* = 15.8 Hz, 1H), 4.15–4.04 (m, 2H), 1.89 (t, *J* = 1.4 Hz, 3H). ^13^C NMR (126 MHz, DMSO-*d*_6_) *δ* 171.65, 169.25, 165.98, 163.29 (d, *J* = 247.4 Hz), 144.38, 139.01, 133.35, 131.77 (d, *J* = 3.2 Hz), 130.35 (d, *J* = 8.8 Hz), 121.50, 116.41 (d, *J* = 21.7 Hz), 93.30, 41.21, 10.58. HRMS (ESI) *m*/*z* calcd for C_16_H_15_FNO_5_ [M + H]^+^: 320.0929; found, 320.0929.

*4-methyl-5-oxo-2,5-dihydrofuran-2-yl(E)-(3-(2-chlorophenyl)acryloyl)glycinate* (**4**), white solid, m.p. 190–191 °C, 64% yield. ^1^H NMR (500 MHz, DMSO-*d*_6_) *δ* 8.77 (t, *J* = 5.9 Hz, 1H), 7.82–7.51 (m, 3H), 7.45–7.40 (m, 2H), 7.36–7.32 (m, 1H), 6.98–6.94 (m, 1H), 6.79 (d, *J* = 15.8 Hz, 1H), 4.18–4.06 (m, 2H), 1.90 (t, *J* = 1.4 Hz, 3H). ^13^C NMR (126 MHz, DMSO-*d*_6_) *δ* 171.65, 169.15, 165.58, 144.38, 135.44, 133.87, 133.37, 132.92, 131.63, 130.48, 128.29, 128.17, 124.67, 93.32, 41.27, 10.59. HRMS (ESI) *m*/*z* calcd for C_16_H_14_ClNNaO_5_ [M + Na]^+^: 358.0453; found, 358.0453.

*4-methyl-5-oxo-2,5-dihydrofuran-2-yl(E)-(3-(3-chlorophenyl)acryloyl)glycinat* (**5**), white solid, m.p. 128–129 °C, 66% yield. ^1^H NMR (500 MHz, DMSO-*d*_6_) *δ* 8.63 (t, *J* = 5.9 Hz, 1H), 7.70–7.55 (m, 2H), 7.49–7.44 (m, 3H), 7.35–7.30 (m, 1H), 6.97–6.94 (m, 1H), 6.79 (d, *J* = 15.9 Hz, 1H), 4.15–4.03 (m, 2H), 1.89 (t, *J* = 1.4 Hz, 3H). ^13^C NMR (126 MHz, DMSO-*d*_6_) *δ* 171.65, 169.18, 165.72, 144.38, 138.66, 137.45, 134.17, 133.36, 131.26, 129.79, 127.83, 126.65, 123.31, 93.31, 41.24, 10.59. HRMS (ESI) *m*/*z* calcd for C_16_H_15_ClNO_5_ [M + H]^+^: 336.0633; found, 336.0632.

*4-methyl-5-oxo-2,5-dihydrofuran-2-yl(E)-(3-(4-chlorophenyl)acryloyl)glycinate* (**6**), white solid, m.p. 177–178 °C, 57% yield. ^1^H NMR (500 MHz, DMSO-*d*_6_) *δ* 8.66 (t, *J* = 5.9 Hz, 1H), 7.65–7.44 (m, 5H), 7.35–7.31 (m, 1H), 6.98–6.93 (m, 1H), 6.74 (d, *J* = 15.9 Hz, 1H), 4.17–4.04 (m, 2H), 1.92–1.85 (m, 3H). ^13^C NMR (126 MHz, DMSO-*d*_6_) *δ* 171.64, 169.22, 165.86, 144.35, 138.87, 134.61, 134.08, 133.37, 129.86, 129.45, 122.39, 93.30, 41.22, 10.58. HRMS (ESI) *m*/*z* calcd for C_16_H_15_ClNO_5_ [M + H]^−^: 336.0633; found, 336.0636.

*4-methyl-5-oxo-2,5-dihydrofuran-2-yl(E)-(3-(2-bromophenyl)acryloyl)glycinate* (**7**), white solid, m.p. 189–190 °C, 41% yield. ^1^H NMR (500 MHz, DMSO-*d*_6_) *δ* 8.75 (t, *J* = 5.9 Hz, 1H), 7.76–7.70 (m, 3H), 7.47 (t, *J* = 7.4 Hz, 1H), 7.37–7.31 (m, 2H), 6.97–6.93 (m, 1H), 6.74 (d, *J* = 15.7 Hz, 1H), 4.17–4.05 (m, 2H), 1.89 (t, *J* = 1.4 Hz, 3H). ^13^C NMR (126 MHz, DMSO-*d*_6_) *δ* 171.65, 169.14, 165.51, 144.38, 138.10, 134.66, 133.70, 133.36, 131.83, 128.83, 128.25, 124.77, 124.74, 93.32, 41.27, 10.60. HRMS (ESI) *m*/*z* calcd for C_16_H_15_BrNO_5_ [M + NH_4_]^+^: 397.0394; found, 397.0393.

*4-methyl-5-oxo-2,5-dihydrofuran-2-yl (E)-(3-(3-bromophenyl)acryloyl)glycinate* (**8**), white solid, m.p. 130–131 °C, 45% yield. ^1^1H NMR (500 MHz, DMSO-*d*_6_) *δ* 8.62 (t, *J* = 5.9 Hz, 1H), 7.85–7.56 (m, 3H), 7.49–7.35 (m, 2H), 7.35–7.29 (m, 1H), 6.97–6.93 (m, 1H), 6.78 (d, *J* = 15.9 Hz, 1H), 4.16–4.04 (m, 2H), 1.89 (s, 3H). ^13^C NMR (126 MHz, DMSO-*d*_6_) *δ* 171.64, 169.18, 165.70, 144.37, 138.58, 137.71, 133.36, 132.68, 131.52, 130.69, 127.03, 123.30, 122.75, 93.31, 41.25, 10.59. HRMS (ESI) *m*/*z* calcd for C_16_H_15_BrNO_5_ [M + H]^+^: 380.0128; found, 380.0128.

*4-methyl-5-oxo-2,5-dihydrofuran-2-yl(E)-(3-(4-chlorophenyl)acryloyl)glycinate* (**9**), white solid, m.p. 183–184 °C, 50% yield. ^1^H NMR (500 MHz, DMSO-*d*_6_) *δ* 8.66 (t, *J* = 5.9 Hz, 1H), 7.66–7.53 (m, 4H), 7.46 (d, *J* = 15.8 Hz, 1H), 7.35–7.31 (m, 1H), 6.97–6.93 (m, 1H), 6.75 (d, *J* = 15.9 Hz, 1H), 4.17–4.01 (m, 2H), 1.89 (t, *J* = 1.3 Hz, 3H). ^13^C NMR (126 MHz, DMSO-*d*_6_) *δ* 171.64, 169.20, 165.84, 144.37, 138.95, 134.43, 133.36, 132.39, 130.11, 123.36, 122.47, 93.30, 41.23, 10.59. HRMS (ESI) *m*/*z* calcd for C_16_H_15_BrNO_5_ [M + H]^+^: 380.0128; found, 380.0128.

*4-methyl-5-oxo-2,5-dihydrofuran-2-yl(E)-(3-(o-tolyl)acryloyl)glycinate* (**10**), white solid, m.p. 127–128 °C, 39% yield. ^1^H NMR (500 MHz, DMSO-*d*_6_) *δ* 8.68 (t, *J* = 5.9 Hz, 1H), 7.77–7.55 (m, 2H), 7.35–7.23 (m, 4H), 6.99–6.92 (m, 1H), 6.62 (d, *J* = 15.7 Hz, 1H), 4.16–4.04 (m, 2H), 2.38 (s, 3H), 1.89 (t, *J* = 1.4 Hz, 3H). ^13^C NMR (126 MHz, DMSO-*d*_6_) *δ* 171.66, 169.28, 166.09, 144.39, 137.61, 137.35, 133.92, 133.36, 131.19, 129.91, 126.90, 126.47, 122.74, 93.31, 41.22, 19.85, 10.59. HRMS (ESI) *m*/*z* calcd for C_17_H_18_NO_5_ [M + H]^+^: 316.1179; found, 316.1182.

*4-methyl-5-oxo-2,5-dihydrofuran-2-yl(E)-(3-(m-tolyl)acryloyl)glycinate* (**11**), white solid, m.p. 168–169 °C, 44% yield. ^1^H NMR (500 MHz, DMSO-*d*_6_) *δ* 8.61 (t, *J* = 5.9 Hz, 1H), 7.47–7.19 (m, 6H), 6.97–6.93 (m, 1H), 6.71 (d, *J* = 15.8 Hz, 1H), 4.15–4.04 (m, 2H), 2.33 (s, 3H), 1.89 (t, *J* = 1.4 Hz, 3H). ^13^C NMR (126 MHz, DMSO-*d*_6_) *δ* 171.65, 169.27, 166.08, 144.39, 140.28, 138.61, 135.06, 133.35, 130.87, 129.33, 128.67, 125.34, 121.44, 93.30, 41.21, 21.37, 10.59. HRMS (ESI) *m*/*z* calcd for C_17_H_18_NO_5_ [M + H]^+^: 316.1179; found, 316.1179.

*4-methyl-5-oxo-2,5-dihydrofuran-2-yl(E)-(3-(p-tolyl)acryloyl)glycinate* (**12**), white solid, m.p. 171–172 °C, 47% yield. ^1^H NMR (500 MHz, DMSO-*d*_6_) *δ* 8.59 (t, *J* = 5.9 Hz, 1H), 7.49 (d, *J* = 8.1 Hz, 2H), 7.44 (d, *J* = 15.8 Hz, 1H), 7.35–7.31 (m, 1H), 7.24 (d, *J* = 8.0 Hz, 2H), 6.96–6.92 (m, 1H), 6.66 (d, *J* = 15.8 Hz, 1H), 4.14–4.04 (m, 2H), 2.33 (s, 3H), 1.89 (t, *J* = 1.4 Hz, 3H). ^13^C NMR (126 MHz, DMSO-*d*_6_) *δ* 171.65, 169.30, 166.19, 144.39, 140.16, 139.97, 133.35, 132.37, 130.03, 128.13, 120.55, 93.29, 41.20, 21.43, 10.59. HRMS (ESI) *m*/*z* calcd for C_17_H_18_NO_5_ [M + H]^+^: 316.1179; found, 316.1179.

*4-methyl-5-oxo-2,5-dihydrofuran-2-yl(E)-(3-(2-methoxyphenyl)acryloyl)glycinate* (**13**), white solid, m.p. 158–159 °C, 42% yield. ^1^H NMR (500 MHz, DMSO-*d*_6_) *δ* 8.63 (t, *J* = 5.9 Hz, 1H), 7.71 (d, *J* = 16.0 Hz, 1H), 7.55 (dd, *J* = 7.7, 1.4 Hz, 1H), 7.43–7.36 (m, 1H), 7.35–7.30 (m, 1H), 7.09 (d, *J* = 8.2 Hz, 1H), 7.00 (t, *J* = 7.5 Hz, 1H), 6.97–6.93 (m, 1H), 6.74 (d, *J* = 16.0 Hz, 1H), 4.14–4.02 (m, 2H), 3.87 (s, 3H), 1.89 (t, *J* = 1.3 Hz, 3H). ^13^C NMR (126 MHz, DMSO-*d*_6_) *δ* 171.66, 169.31, 166.45, 158.12, 144.40, 135.25, 133.35, 131.57, 128.53, 123.48, 121.96, 121.19, 112.20, 93.29, 56.05, 41.20, 10.59. HRMS (ESI) *m*/*z* calcd for C_17_H_18_NO_6_ [M + H]^+^: 332.1129; found, 332.1128.

*4-methyl-5-oxo-2,5-dihydrofuran-2-yl(E)-(3-(3-methoxyphenyl)acryloyl)glycinate* (**14**), white solid, m.p. 117–118 °C, 52% yield. ^1^H NMR (500 MHz, DMSO-*d*_6_) *δ* 8.61 (t, *J* = 5.9 Hz, 1H), 7.49–7.30 (m, 3H), 7.21–7.13 (m, 2H), 6.99–6.94 (m, 2H), 6.73 (d, *J* = 15.8 Hz, 1H), 4.17–4.01 (m, 2H), 3.79 (s, 3H), 1.89 (t, *J* = 1.4 Hz, 3H). ^13^C NMR (126 MHz, DMSO-*d*_6_) *δ* 171.65, 169.25, 166.02, 160.07, 144.39, 140.13, 136.56, 133.35, 130.48, 121.96, 120.51, 116.00, 113.21, 93.30, 55.61, 41.21, 10.59. HRMS (ESI) *m*/*z* calcd for C_17_H_18_NO_6_ [M + H]^+^: 332.1129; found, 332.1129.

*4-methyl-5-oxo-2,5-dihydrofuran-2-yl(E)-(3-(4-methoxyphenyl)acryloyl)glycinate* (**15**), white solid, m.p. 185–186 °C, 48% yield. ^1^H NMR (500 MHz, DMSO-*d*_6_) *δ* 8.54 (t, *J* = 5.9 Hz, 1H), 7.55 (d, *J* = 8.7 Hz, 2H), 7.43 (d, *J* = 15.8 Hz, 1H), 7.34–7.31 (m, 1H), 6.99 (d, *J* = 8.8 Hz, 2H), 6.96–6.93 (m, 1H), 6.57 (d, *J* = 15.8 Hz, 1H), 4.14–4.02 (m, 2H), 3.79 (s, 3H), 1.89 (t, *J* = 1.4 Hz, 3H). ^13^C NMR (126 MHz, DMSO-*d*_6_) *δ* 171.66, 169.35, 166.34, 160.98, 144.40, 139.92, 133.34, 129.77, 127.68, 119.08, 114.88, 93.29, 55.74, 41.18, 10.58. HRMS (ESI) *m*/*z* calcd for C_17_H_18_NO_6_ [M + H]^+^: 332.1129; found, 332.1128.

*4-methyl-5-oxo-2,5-dihydrofuran-2-ylcinnamoylglycinate* (**16**), white solid, m.p. 151–152 °C, 64% yield. ^1^H NMR (500 MHz, DMSO-*d*_6_) *δ* 8.65 (t, *J* = 5.9 Hz, 1H), 7.62–7.57 (m, 2H), 7.51–7.37 (m, 4H), 7.35–7.32 (m, 1H), 6.98–6.92 (m, 1H), 6.73 (d, *J* = 15.8 Hz, 1H), 4.16–4.03 (m, 2H), 1.89 (t, *J* = 1.3 Hz, 3H). ^13^C NMR (126 MHz, DMSO-*d*_6_) *δ* 171.65, 169.26, 166.04, 144.39, 140.20, 135.12, 133.35, 130.17, 129.44, 128.15, 121.62, 93.30, 41.21, 10.59. HRMS (ESI) *m*/*z* calcd for C_16_H_16_NO_5_ [M + H]^+^: 302.1023; found, 302.1023.

### 3.3. Parasitic Weed Seed Germination Bioassays

Parasitic weed seed germination bioassay was conducted with reference to the previously described method [32]. The seeds were sterilized with 1% (*w*/*w*) sodium hypochlorite (NaClO) for 2 min and then soaked in 75% (*v*/*v*) ethanol for another 2 min. After the surface sterilization, all of the seeds were thoroughly rinsed with sterile water and finally dried in air above a clean bench. The glass fiber filter paper discs (10 mm, Grade GF/D, Whatman, Cytiva, Wilmington, Delaware, USA) were placed in a Petri dish containing a filter paper ring wetted with sterile water, and the pretreated seeds of *O. aegyptiaca* (about 30–80 seeds) were spread onto each. The Petri dishes were sealed and preincubated in the dark at 25 °C for 7 days. Then, the preconditioned seeds were treated with 50 μL of each tested compound solution with indicated concentration, and the content of DMSO of the solution was less than 1% (*v*/*v*). Each treatment was carried out five times. After application, the sealed Petri dishes were kept in the dark and incubated at 25 °C for 7 days. Finally, the germinated seeds were counted under a binocular microscope and the germination rate (%) was calculated. The sterile water (containing 1% DMSO) was used as a negative control, and the *rac*-GR24 was included as a positive control. The EC_50_ (half-maximal concentration) values of the compounds were calculated using the GraphPad Prism 8.0 program.

### 3.4. Arabidopsis Primary Root Elongation, Lateral Root Formation, and Root Hair Elongation Assay

Considering the previously reported procedures [36,37], *Arabidopsis* seeds were sterilized with 70% ethanol for 1 min and 1% sodium hypochlorite solution for another 15 min and were then cleaned five times with sterile water. Half-strength MS (Murashige–Skoog) was prepared with 0.8% agar, 1% sucrose, and 0.5 g L^−1^ MES, then the pH was adjusted to 5.7. After that, the sterilized *Arabidopsis* seeds were sown on the sterilized media supplemented with tested compounds. Each medium was incubated at 4 °C in the dark for 3 days and then vertically placed in the illumination box, at 22 °C with 60% relative humidity. All of the plants were maintained under 16 h light/8 h darkness for 8 days. Subsequently, the *Arabidopsis* seedlings were scanned, and the primary root length, number of lateral roots, and root hair length of each were measured using publicly available ImageJ software (https://imagej.nih.gov/ij/index.html, accessed on 11 December 2022). The inhibition rate of root growth was calculated according to the following equation:

$$I=\frac{d_{0}-d}{d_{0}}\times 100\%$$

where *I* is the inhibition rate, and *d*_0_ and *d* are the average lengths of the *Arabidopsis* root in the control and in the presence of the target compounds, respectively. The numbers of lateral roots and the root hair lengths of *Arabidopsis* were subjected to one-way analysis of variance (ANOVA) followed by Duncan’s test at a significance of *p* < 0.05 using SPSS Statistics version 24.0.

### 3.5. Molecular Docking

The molecular docking study was performed using Sybyl 7.3 software. The crystal structure of rice DWARF14 (OsD14; PDB: 5DJ5) was acquired from the RCSB Protein Data Bank and further used for the docking study. The docking results were visualized using PyMol (version 1.9.0) (http://www.pymol.org/, accessed on 5 January 2023) and Discovery Studio 2016 Client (for 2D interaction). 

### 3.6. Stability Test

The stability test was performed with reference to the previously described method [35]. Compound **6** was dissolved in acetonitrile to give a stock solution (10 mM). Then, 10 μL of the previous solution was added to 990 μL of phosphate buffer (pH 8), phosphate citrate buffer (pH 7), and phosphate citrate buffer (pH 5), respectively. All of the samples were incubated at 25 °C. The stability of compound **6** was measured by high-performance liquid chromatography in tandem with a triple quadrupole mass spectrometry (HPLC-MS/MS) after 1, 2, 3, 4, 5, 6, and 7 days. The stability test of the control GR24 was conducted in a similar manner. 

## 4. Conclusions

In summary, a series of novel SL mimics derived from Cinnamic amide were obtained through a relatively short number of synthetic steps for the first time. Bioassay indicated that compound **6** encouraged potent seed germination activity in the *O. aegyptiaca*, which was expected to be used for the control of parasitic weeds. In addition, it was surprising that compound **6** not only exhibited strong inhibitory activity on the primary root elongation and lateral root formation of *Arabidopsis*, but also displayed significant activity in promoting root hair elongation. The structure–activity relationship revealed that the introduction of an electron-withdrawing substituent on the para position of the benzene ring was beneficial for bioactivity; in particular, the introduction of Cl was superior to F and Br. Morphological experiments on *Arabidopsis max2-1* mutants revealed that **6** possessed SL-like physiological functions, which affected *Arabidopsis* lateral root formation and root hair elongation in a MAX2-dependent manner. Furthermore, molecular docking studies demonstrated that the carbonyl group on the butenolide moieties of **6** reached toward the key residue Ser 97 to form a hydrogen bond in the active site of OsD14, and its binding conformation was similar to that of GR24. These results suggest that **6** is a potential SL mimic for agricultural application. This work provides valuable clues for the rational design and optimization of new hybrid-type SL mimics.

## Figures and Tables

**Table 1:** Seed Germination Activity towards *O. aegyptiaca* ^a^.

Compds	R	Germination Rate (%) ^a^			EC_50_ (M)
		10^−5^ (M)	10^−6^ (M)	10^−7^ (M)	
**1**	2-F	51.7 ± 8.8	35.6 ± 0.8	26.0 ± 2.2	n.d
**2**	3-F	56.0 ± 6.2	44.1 ± 9.2	30.8 ± 8.0	n.d
**3**	4-F	52.7 ± 5.7	47.9 ± 3.1	40.1 ± 6.4	1.15 × 10^−6^
**4**	2-Cl	57.3 ± 5.7	42.5 ± 7.6	27.8 ± 5.2	1.72 × 10^−6^
**5**	3-Cl	80.5 ± 7.9	81.6 ± 8.0	53.3 ± 4.2	8.02 × 10^−8^
**6**	4-Cl	93.7 ± 7.1	90.7 ± 4.3	87.1 ± 4.9	2.36 × 10^−8^
**7**	2-Br	92.4 ± 3.4	68.3 ± 4.9	63.5 ± 7.8	4.47 × 10^−8^
**8**	3-Br	81.1 ± 8.3	53.8 ± 0.2	56.3 ± 2.4	6.05 × 10^−8^
**9**	4-Br	53.4 ± 6.5	23.9 ± 4.8	30.1 ± 7.2	n.d
**10**	2-CH_3_	50.2 ± 6.9	25.9 ± 6.5	27.6 ± 7.2	n.d
**11**	3-CH_3_	78.0 ± 5.0	25.0 ± 6.1	26.3 ± 9.0	3.42 × 10^−6^
**12**	4-CH_3_	21.6 ± 8.1	12.1 ± 3.0	22.2 ± 8.9	n.d
**13**	2-OCH_3_	68.6 ± 7.0	39.0 ± 5.4	40.0 ± 6.3	n.d
**14**	3-OCH_3_	23.1 ± 6.6	14.6 ± 5.0	16.6 ± 5.5	n.d
**15**	4-OCH_3_	31.9 ± 6.0	29.7 ± 6.3	27.2 ± 6.7	n.d
**16**	H	44.7 ± 2.0	39.4 ± 2.9	25.5 ± 7.7	n.d
**GR24**	-	95.3 ± 1.7	96.5 ± 3.2	97.6 ± 1.7	3.09 × 10^−9^

^a^ The seed germination rate for the negative control (distilled water) was lower than 5%. n.d: not determined.

**Table 2:** Effect of Compounds **1**–**16** on primary root elongation of *Arabidopsis* (Columbia-0, Col-0).

Compds	R	Inhibition Rate (%)			IC_50_ (μM)
		1 (μM)	50 (μM)	100 (μM)	
**1**	2-F	−17.4 ± 2.1	37.0 ± 1.9	74.5 ± 1.7	62.96
**2**	3-F	−10.7 ± 1.8	63.0 ± 1.9	74.8 ± 2.8	42.91
**3**	4-F	−0.4 ± 1.3	65.8 ± 3.2	76.0 ± 3.2	38.80
**4**	2-Cl	−3.5 ± 7.6	57.9 ± 4.0	72.6 ± 2.4	47.02
**5**	3-Cl	−5.3 ± 0.7	30.7 ± 2.1	64.9 ± 2.8	74.13
**6**	4-Cl	2.7 ± 4.3	83.2 ± 0.6	91.3 ± 2.1	25.21
**7**	2-Br	8.0 ± 4.4	52.8 ± 5.5	68.6 ± 2.4	47.67
**8**	3-Br	3.3 ± 5.0	48.6 ± 6.1	76.5 ± 7.0	49.41
**9**	4-Br	−2.6 ± 7.7	47.3 ± 5.8	84.1 ± 2.5	52.66
**10**	2-CH_3_	−6.9 ± 6.2	67.3 ± 2.0	72.8 ± 3.1	40.08
**11**	3-CH_3_	0.3 ± 4.4	53.5 ± 4.9	69.1 ± 2.2	49.40
**12**	4-CH_3_	1.3 ± 0.8	71.2 ± 2.8	76.2 ± 2.3	37.37
**13**	2-OCH_3_	8.4 ± 1.6	25.3 ± 1.3	72.4 ± 0.8	71.91
**14**	3-OCH_3_	−1.3 ± 2.5	64.1 ± 0.4	67.0 ± 2.3	44.84
**15**	4-OCH_3_	2.3 ± 0.5	66.5 ± 5.6	70.7 ± 2.8	41.24
**16**	H	2.3 ± 1.8	49.9 ± 4.1	66.5 ± 1.2	54.08
**GR24**	-	3.0 ± 4.3	94.4 ± 1.2	96.6 ± 0.9	9.03

## Data Availability

All data presented in this study are available in the article and in the Supplementary Materials.

## Supplementary Materials

The following supporting information can be downloaded at: https://www.mdpi.com/article/10.3390/ijms24129967/s1.
