# Supplementary material for: Discovery of Novel Hybrid-Type Strigolactone Mimics Derived from Cinnamic Amide
Source: Int J Mol Sci. 2023 Jun 9;24(12):9967. doi: 10.3390/ijms24129967 (PMC10298333; doi:10.3390/ijms24129967)

Supplementary Materials

Discovery of Novel Hybrid-type Strigolactone Mimics Derived from Cinnamic amide

Chunying Wang [1], Bingbo Guo [2], Zhaokai Yang [2], Lin Du [1], Chunxin Yu [3], Yuyi Zhou [1], Hanqing Zhao [3], Ye Wang [3], and Liusheng Duan [1,3,*]

[1] State Key Laboratory of Plant Physiology and Biochemistry, Engineering Research Center of Plant Growth Regulator, Ministry of Education & College of Agronomy and Biotechnology, China Agricultural University, Beijing 100193, China

[2] Innovation Center of Pesticide Research, Department of Applied Chemistry, College of Science, China Agricultural University, Beijing 100193, China

[3] College of Plant Science and Technology, Beijing University of Agriculture, Beijing 102206, China

*Correspondence: duanlsh@cau.edu.cn (L.D.); Tel.: +86-10-62731301

Content

1. **Figure S1.** Stability test of compound **6** and GR24 2

2. ^{1}H NMR and ^{13}C NMR spectra of compounds **1-16**..3

3. HRMS spectra of target compounds **1-16**..19

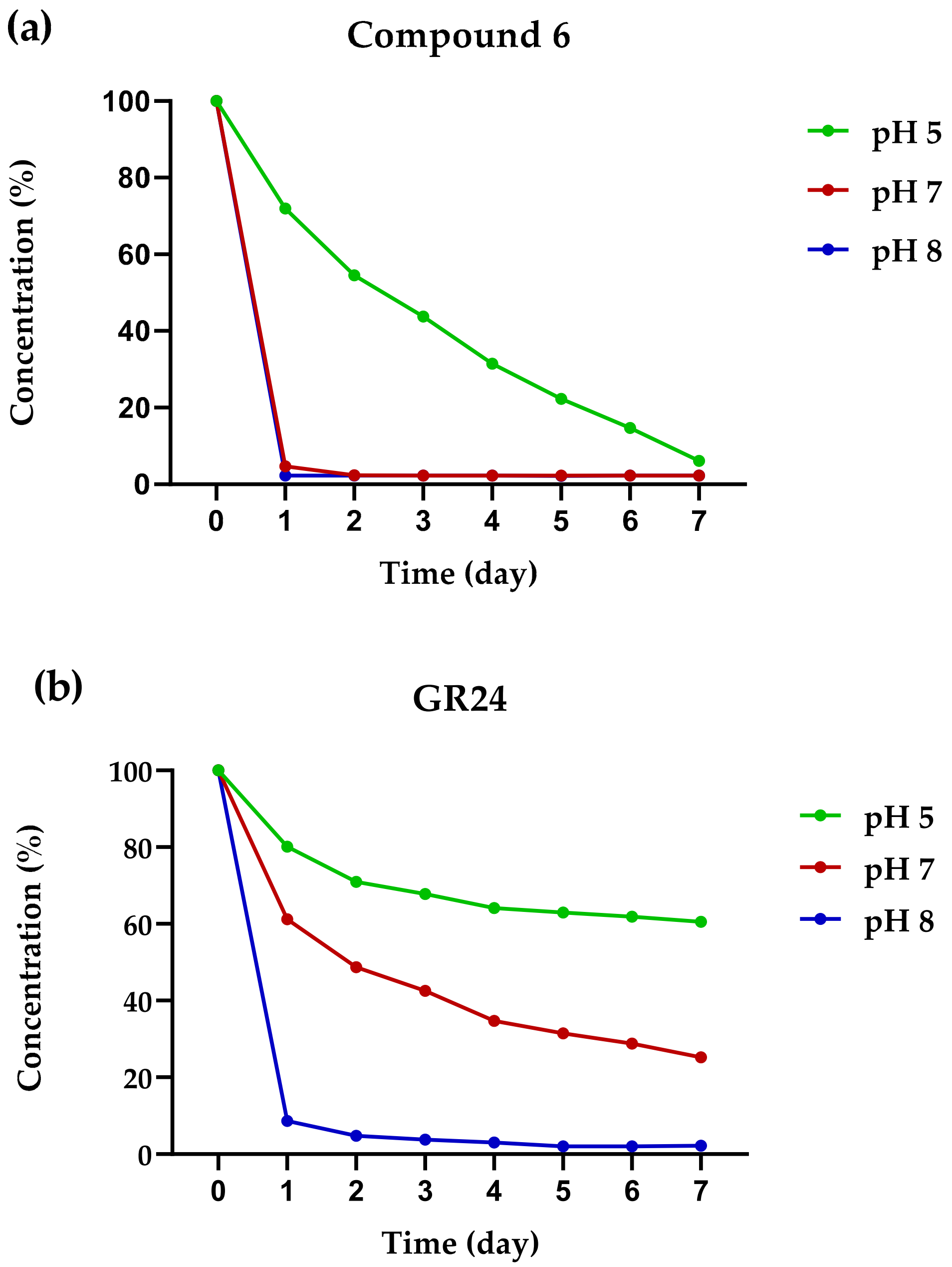

Figure S1. The stability test of compound **6** and GR24. (a) The stability test of compound **6**. (b) The stability test of GR24.

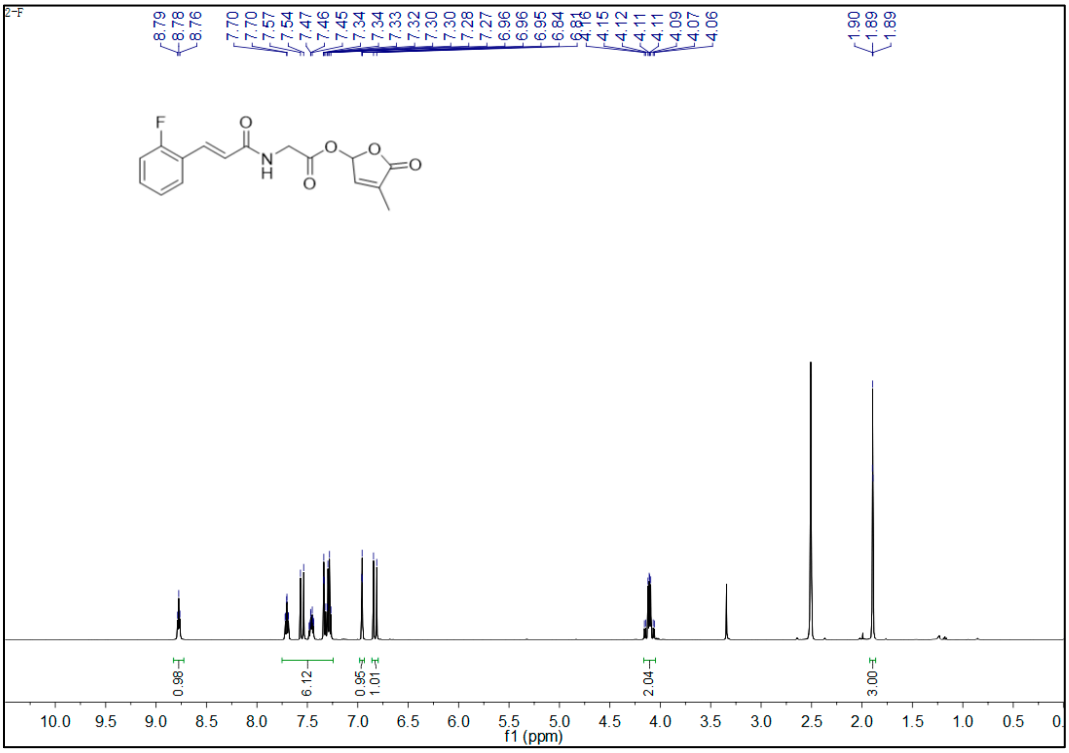

^{1}H NMR spectra of compound **1**

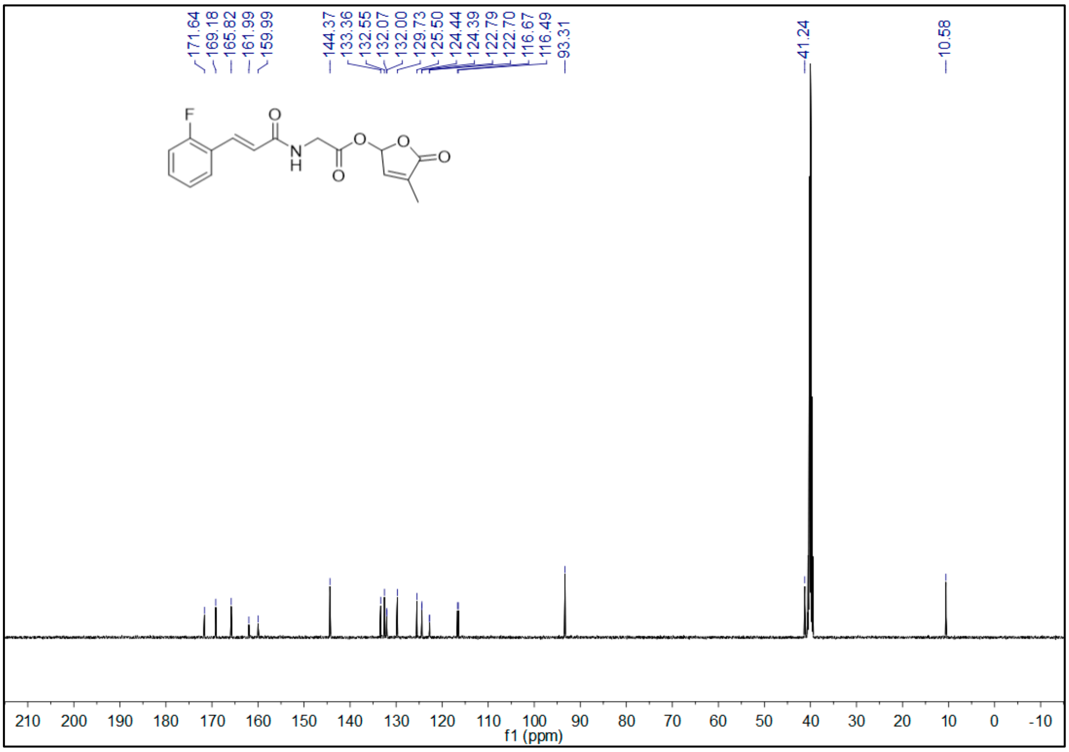

^{13}C NMR spectra of compound **1**

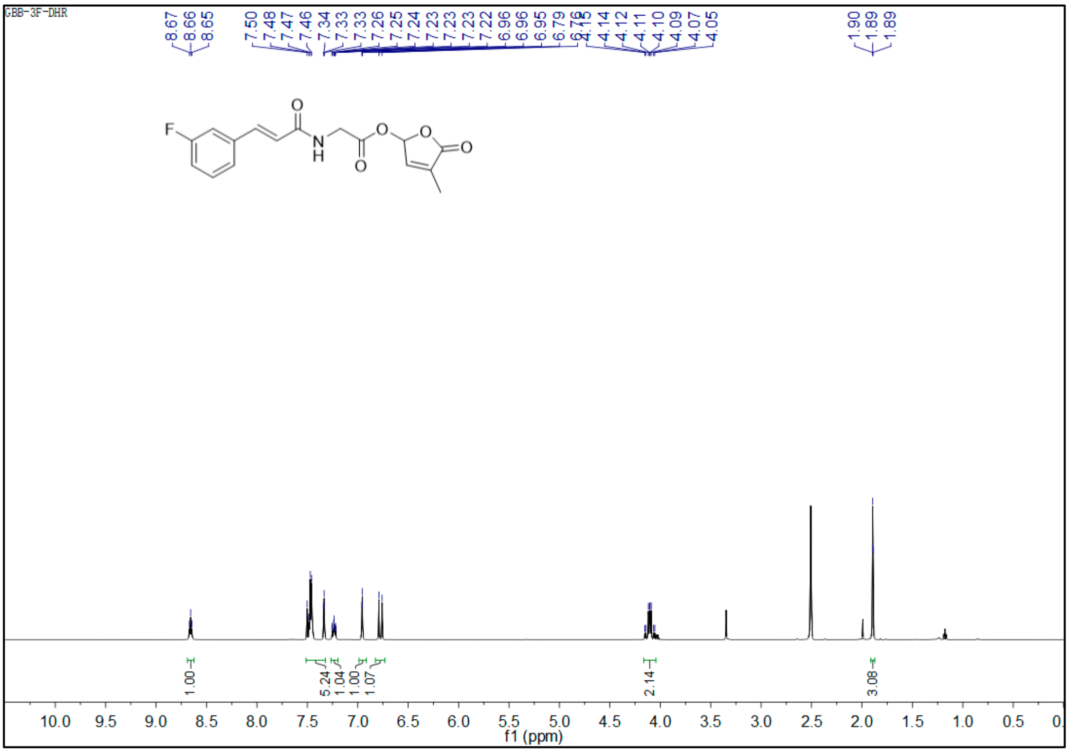

^{1}H NMR spectra of compound **2**

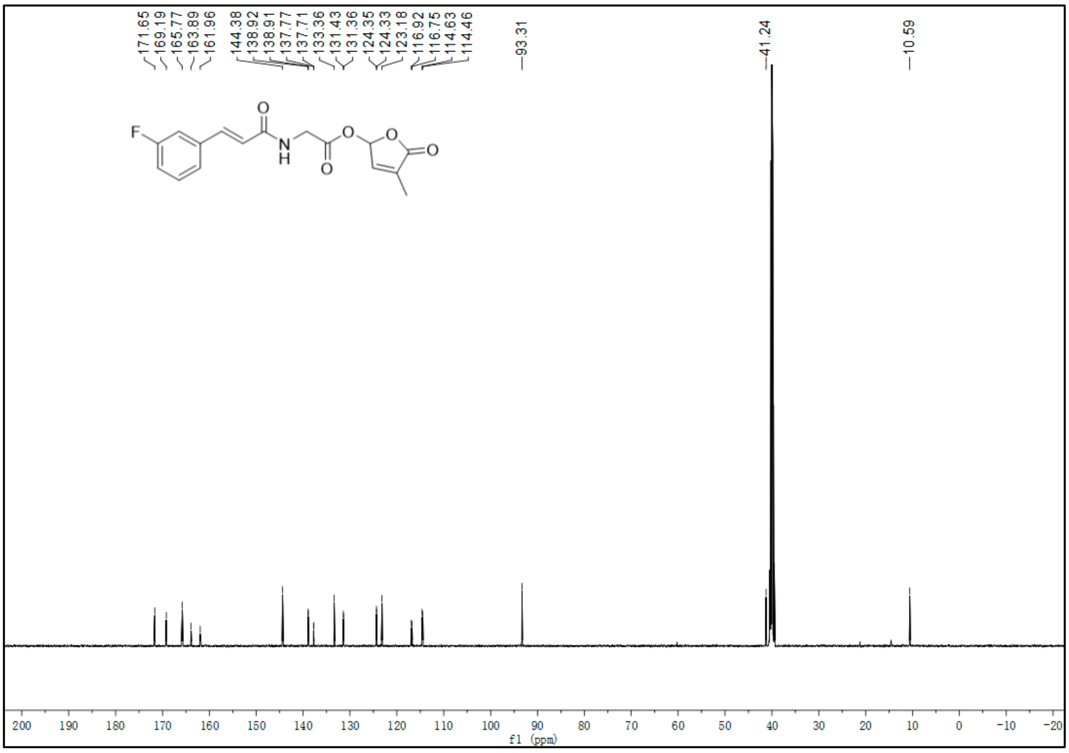

^{13}C NMR spectra of compound **2**

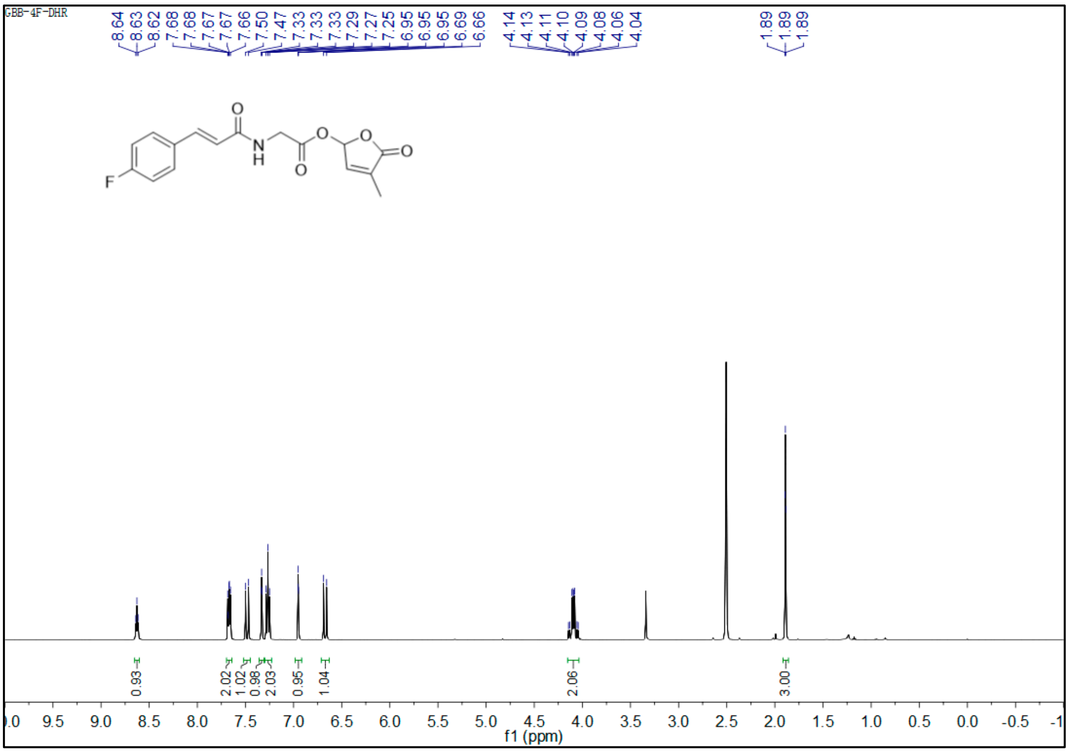

^{1}H NMR spectra of compound **3**

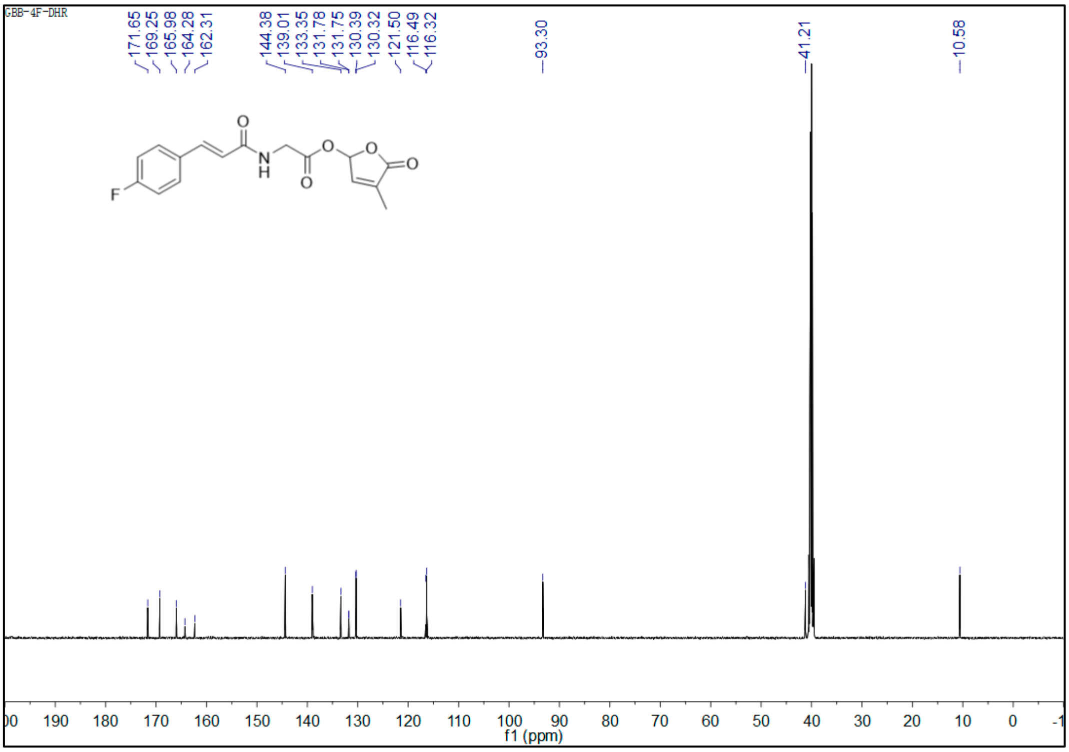

^{13}C NMR spectra of compound **3**

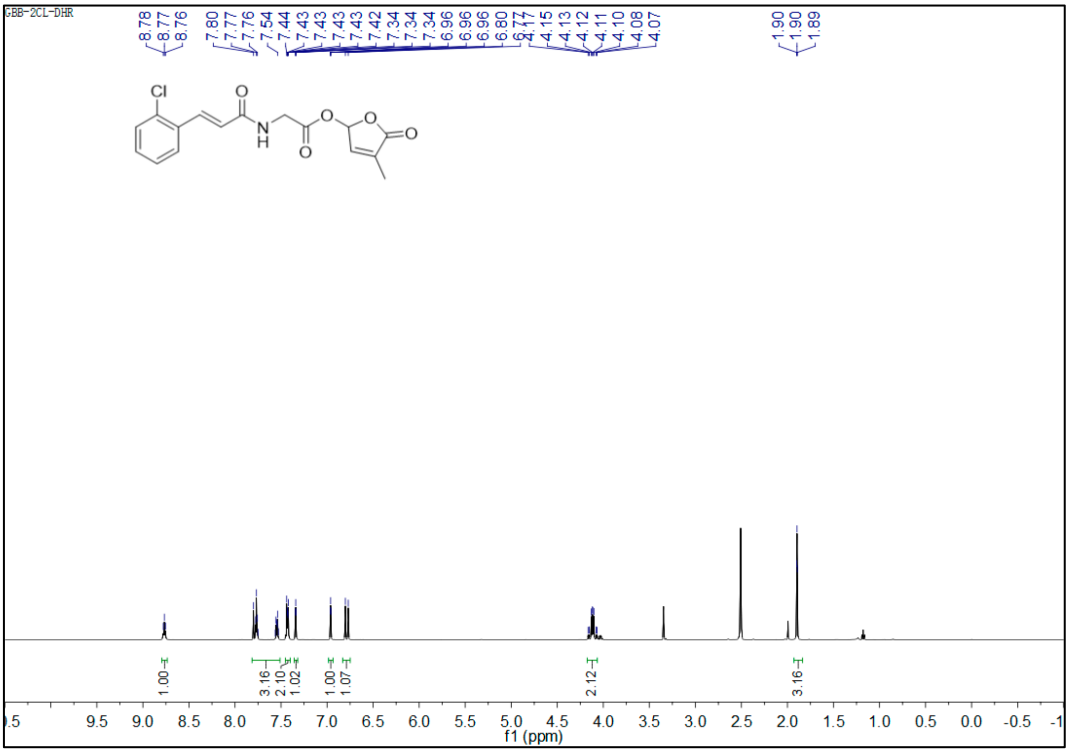

¹H NMR spectra of compound **4**

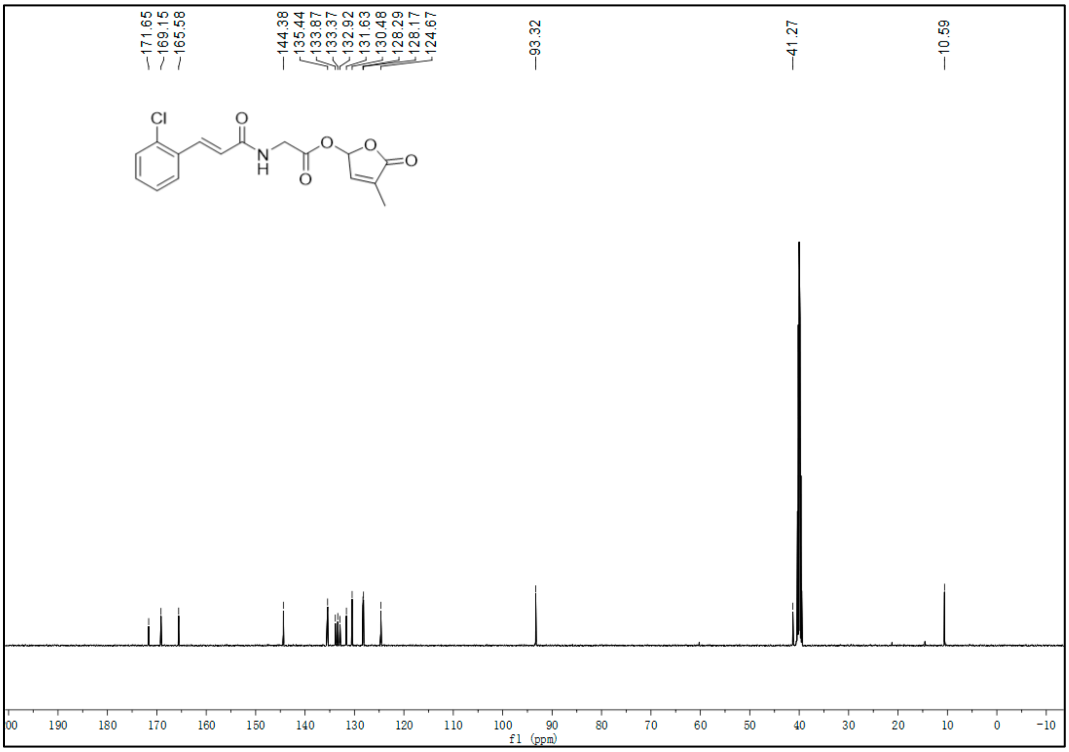

¹³C NMR spectra of compound **4**

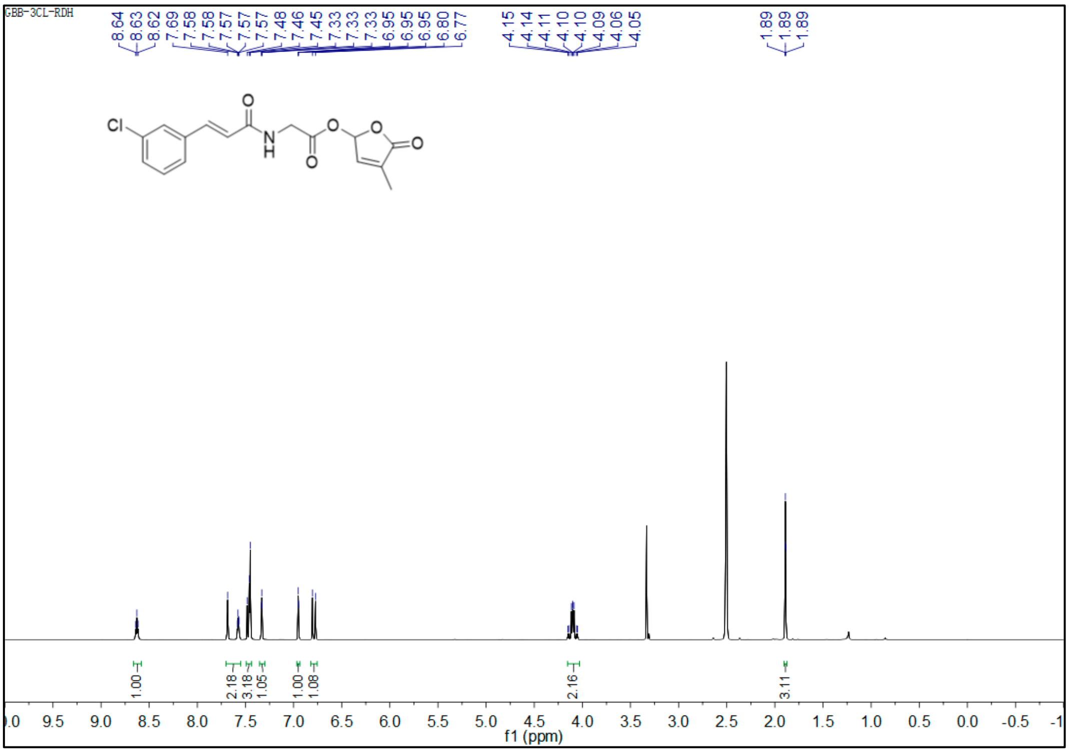

^{1}H NMR spectra of compound **5**

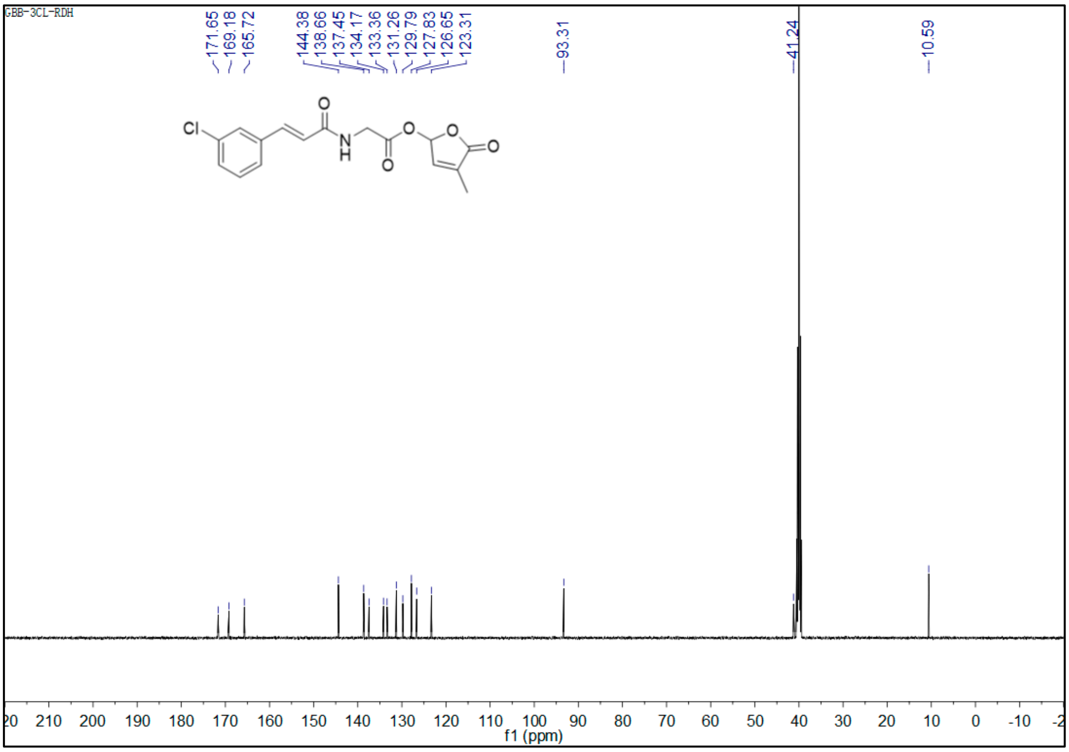

^{13}C NMR spectra of compound **5**

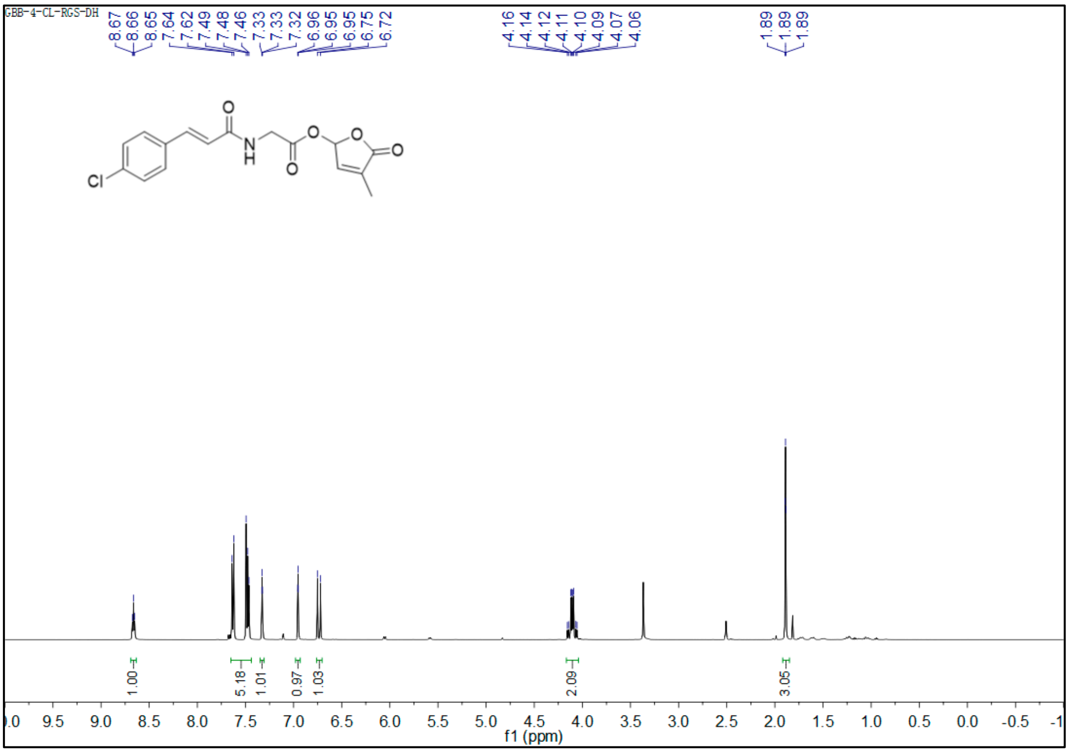

^{1}H NMR spectra of compound **6**

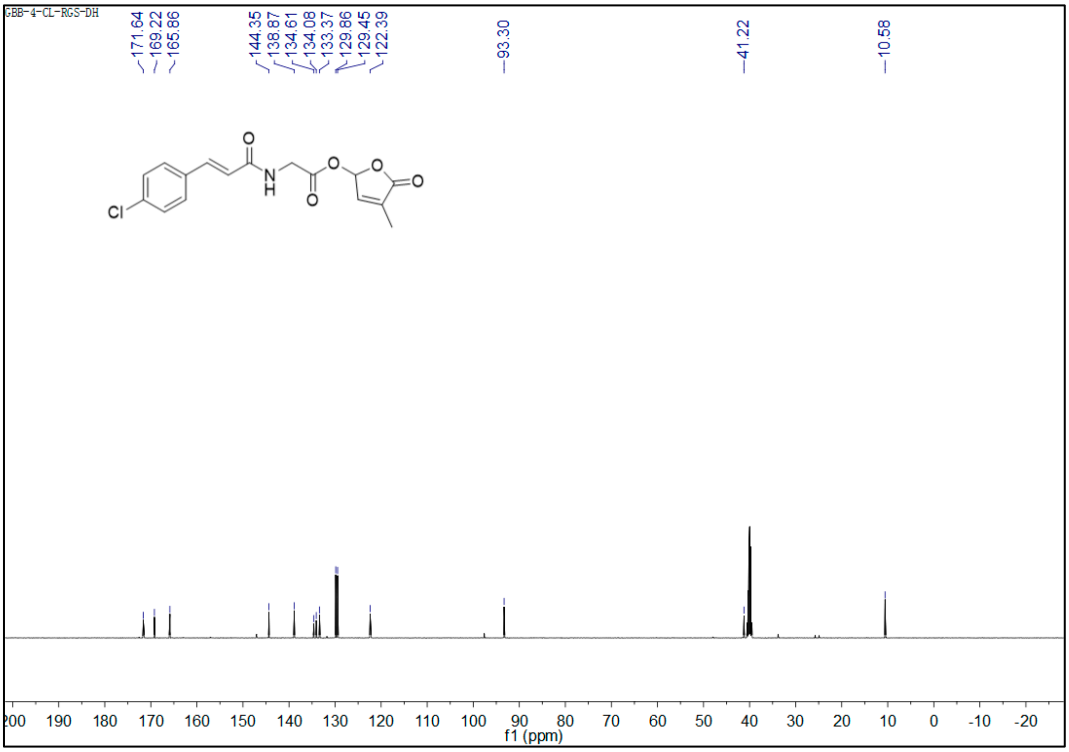

^{13}C NMR spectra of compound **6**

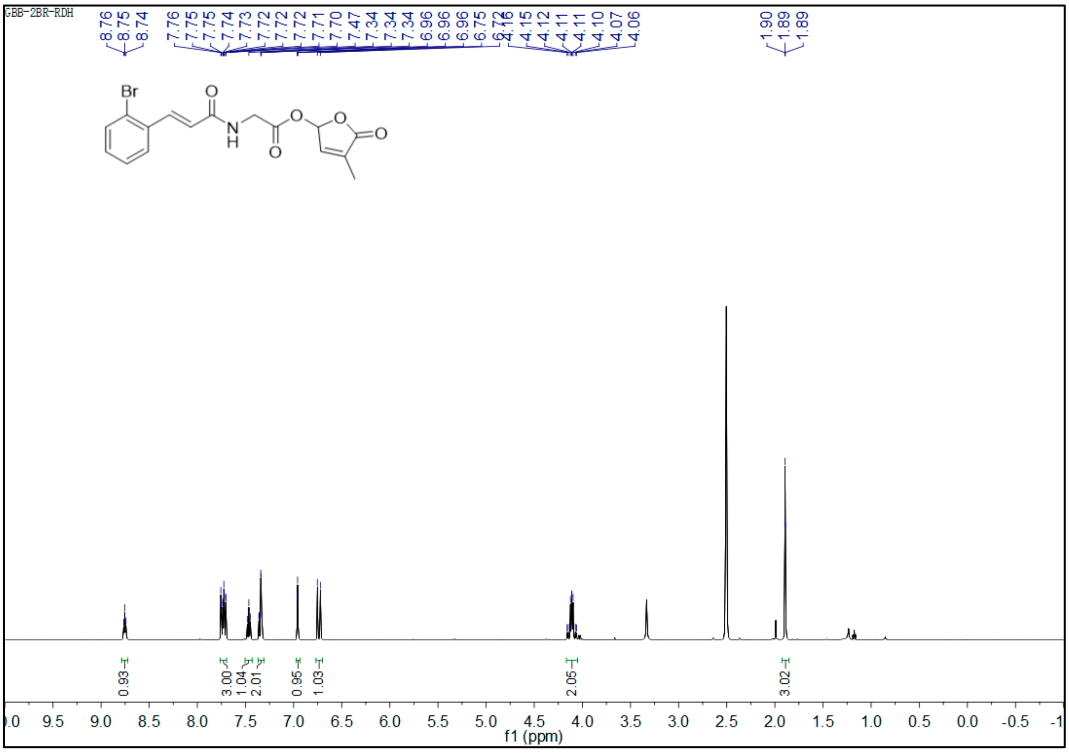

^{1}H NMR spectra of compound **7**

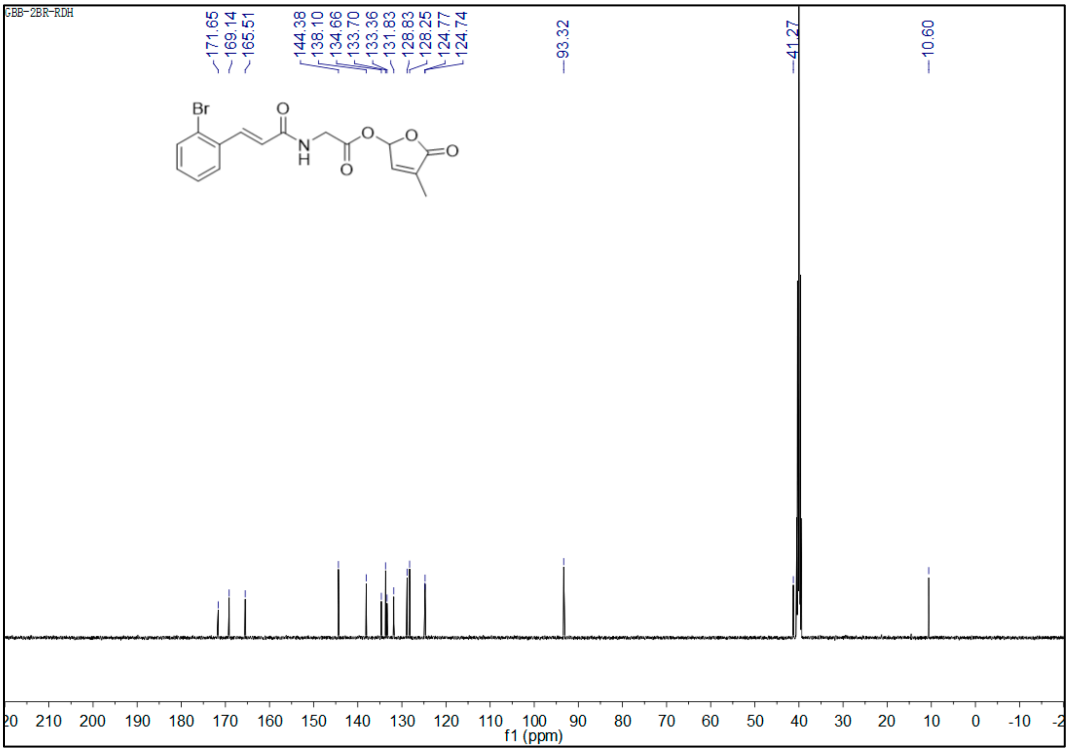

^{13}C NMR spectra of compound **7**

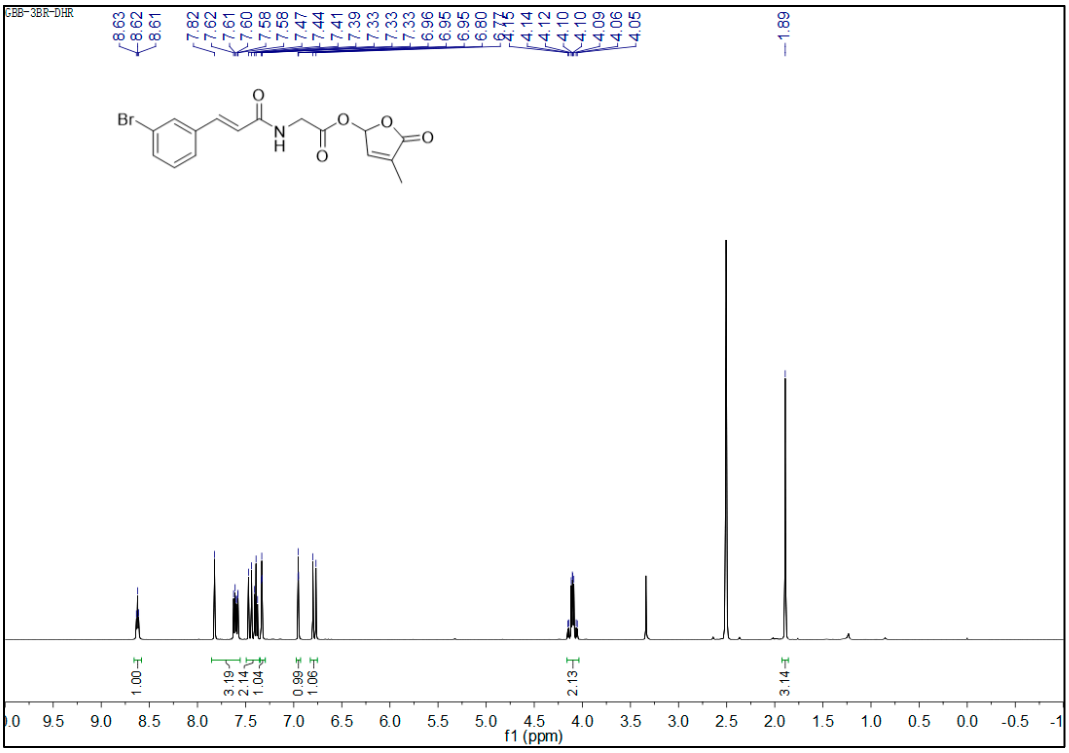

^{1}H NMR spectra of compound 8

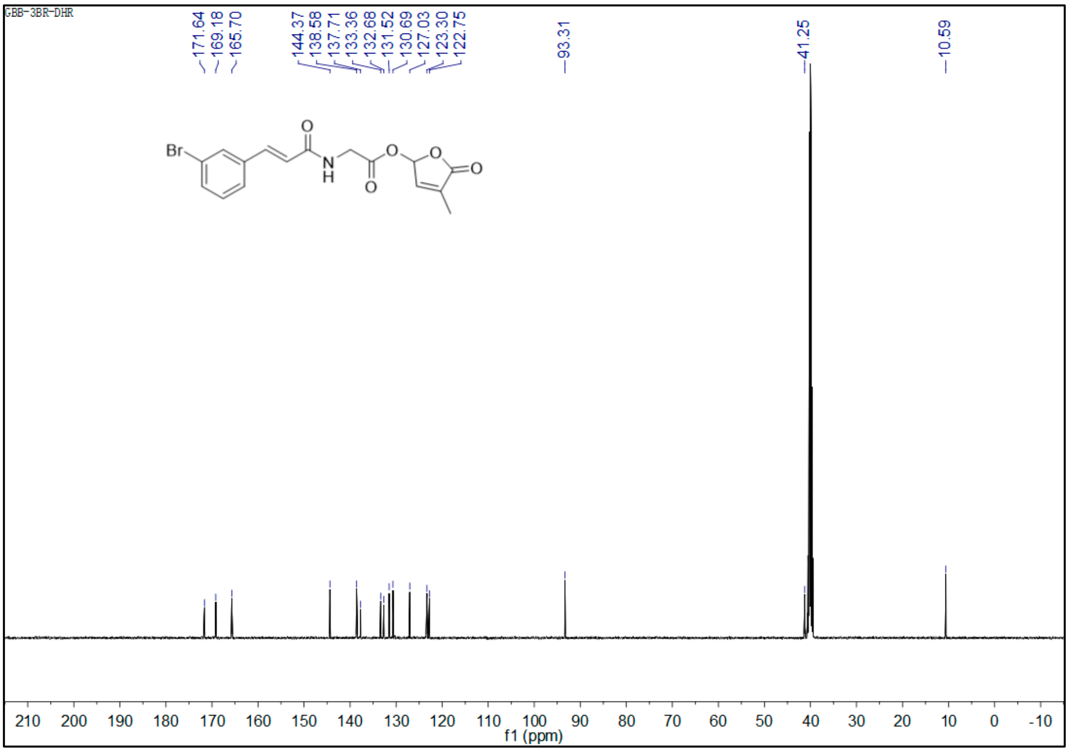

^{13}C NMR spectra of compound **8**

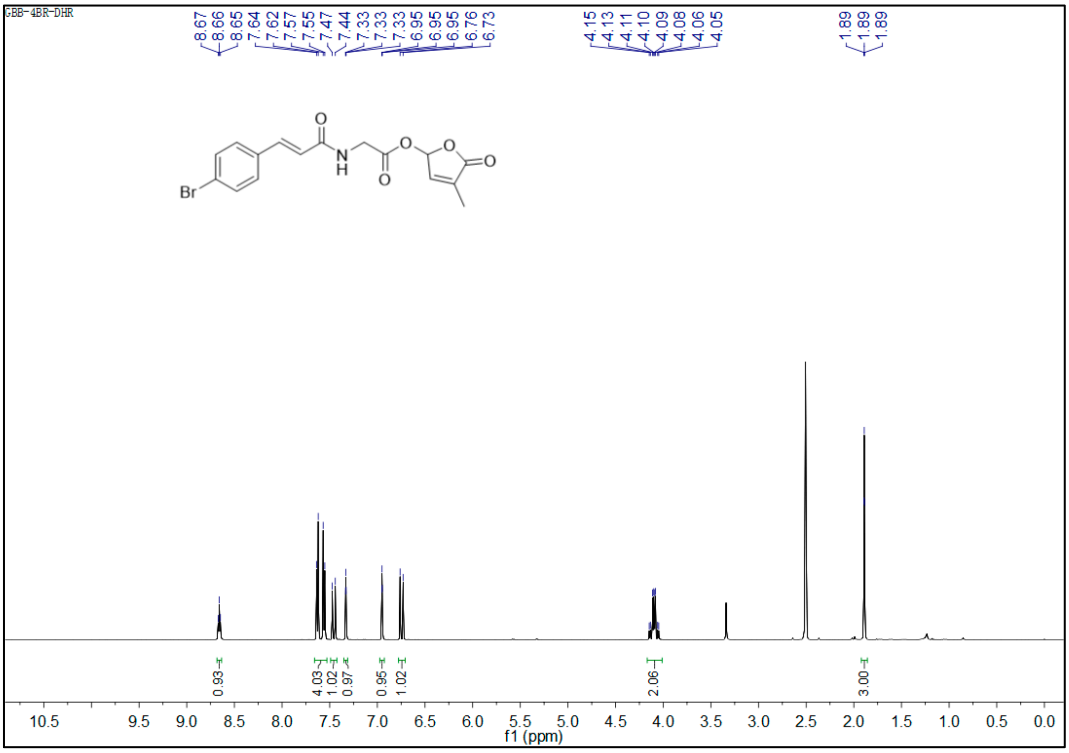

^{1}H NMR spectra of compound **9**

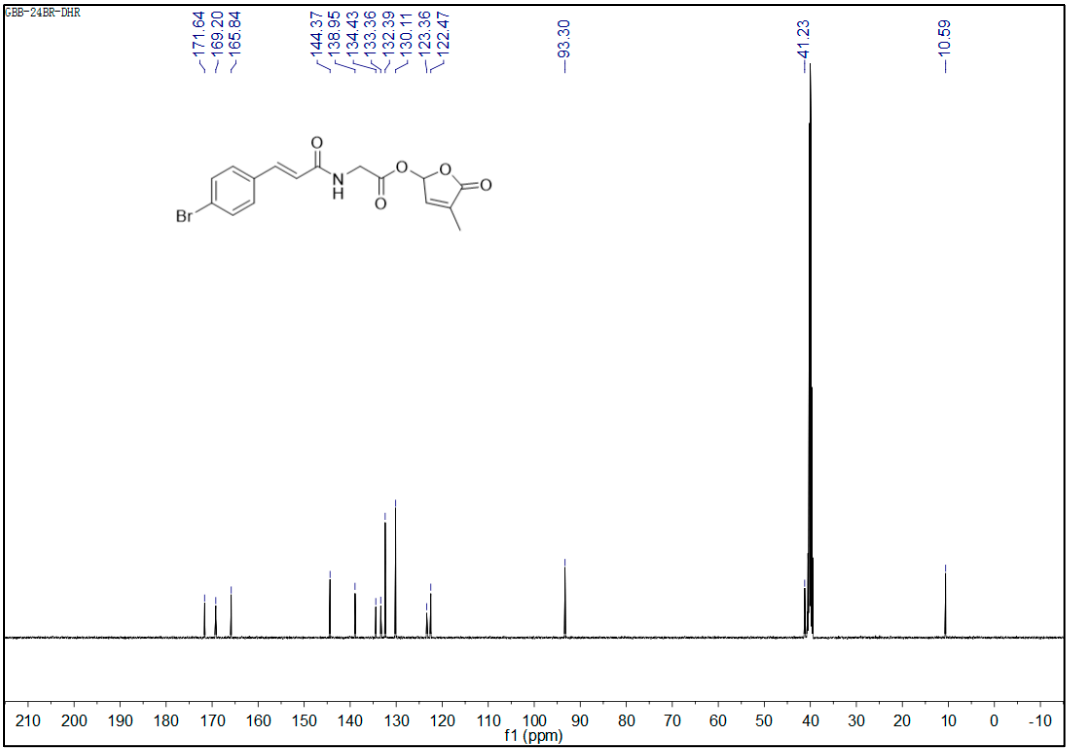

^{13}C NMR spectra of compound **9**

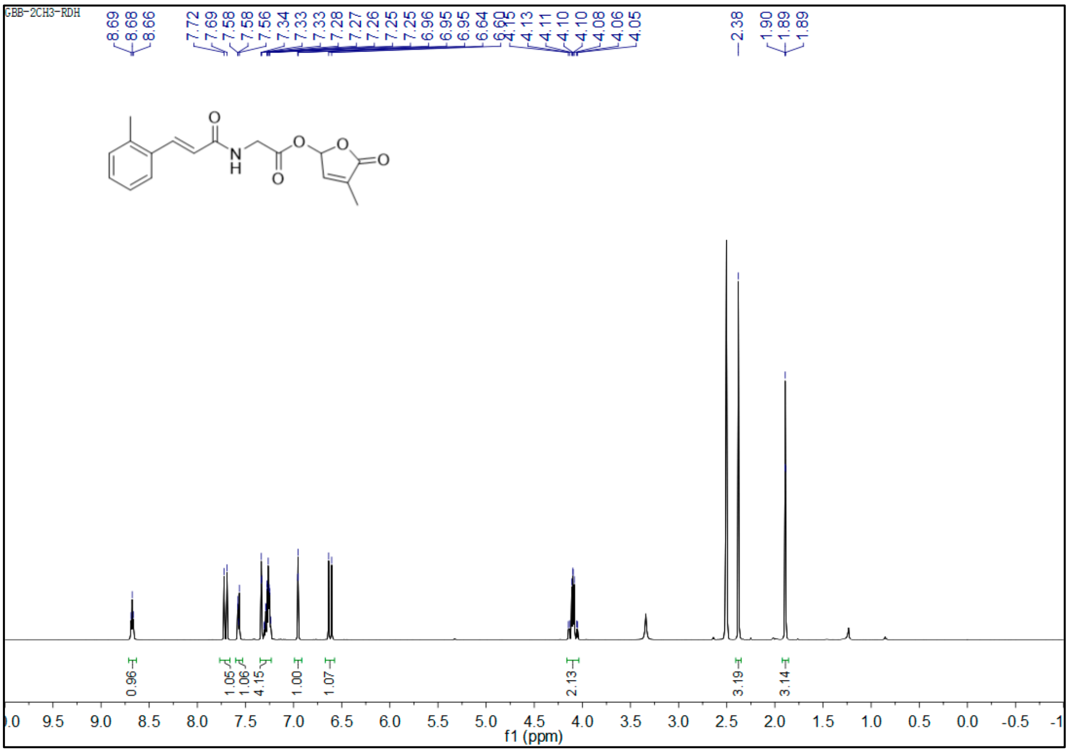

^{1}H NMR spectra of compound **10**

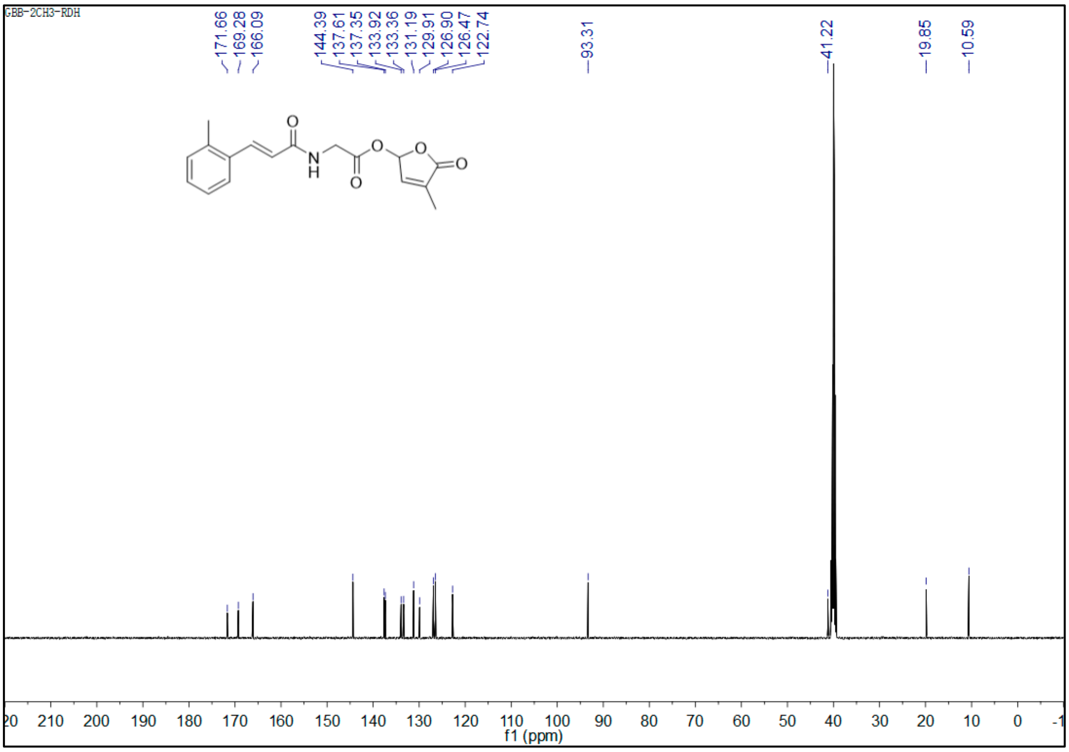

^{13}C NMR spectra of compound **10**

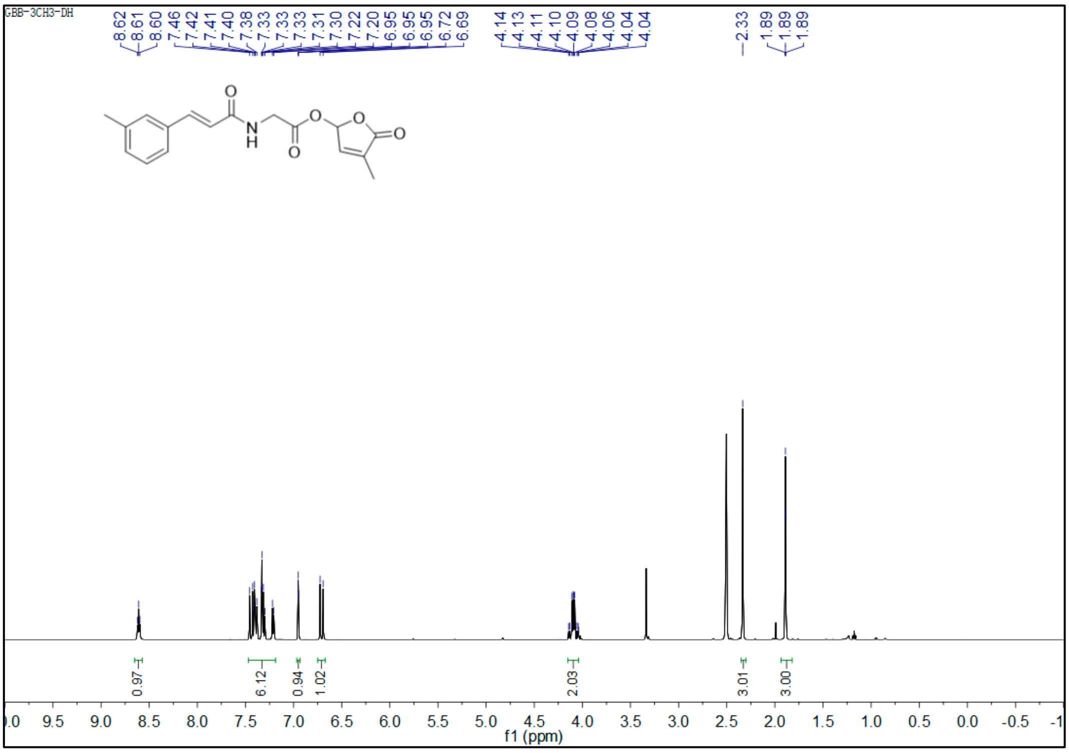

^{1}H NMR spectra of compound **11**

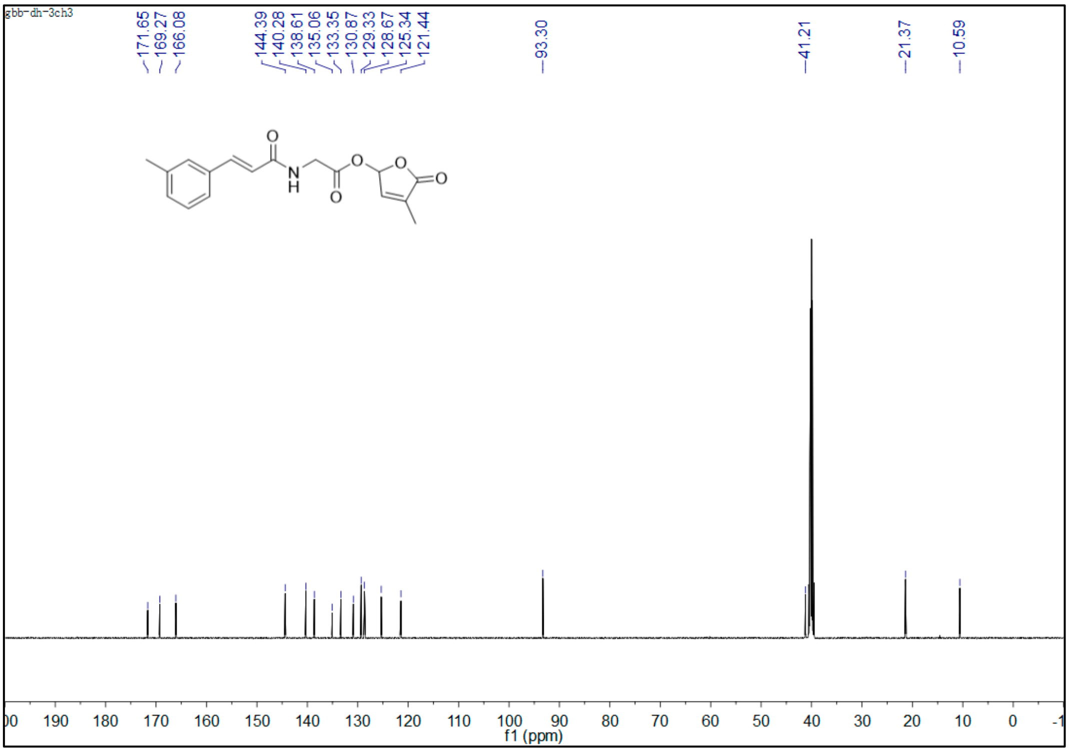

^{13}C NMR spectra of compound **11**

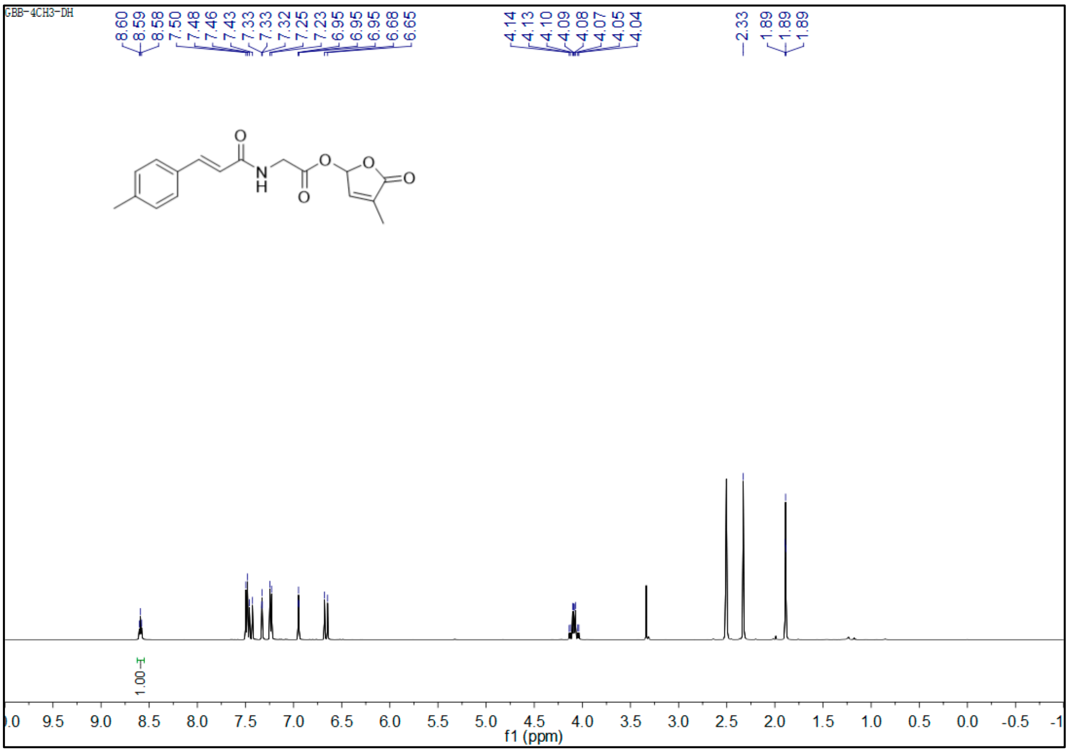

^{1}H NMR spectra of compound **12**

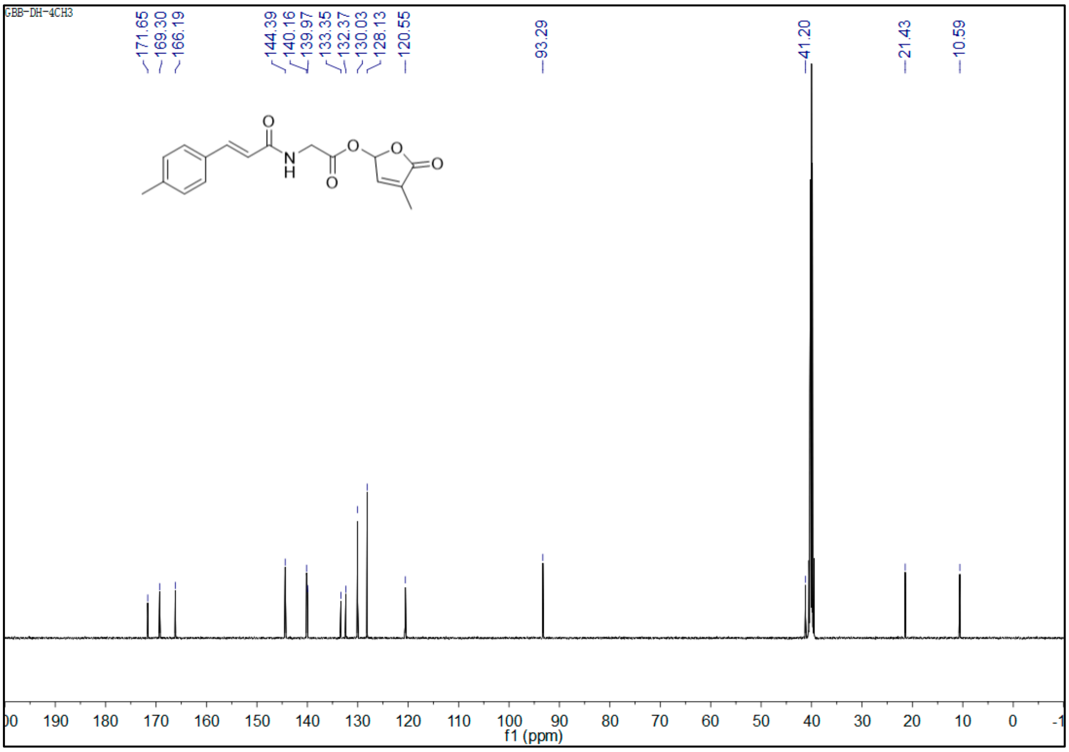

^{13}C NMR spectra of compound **12**

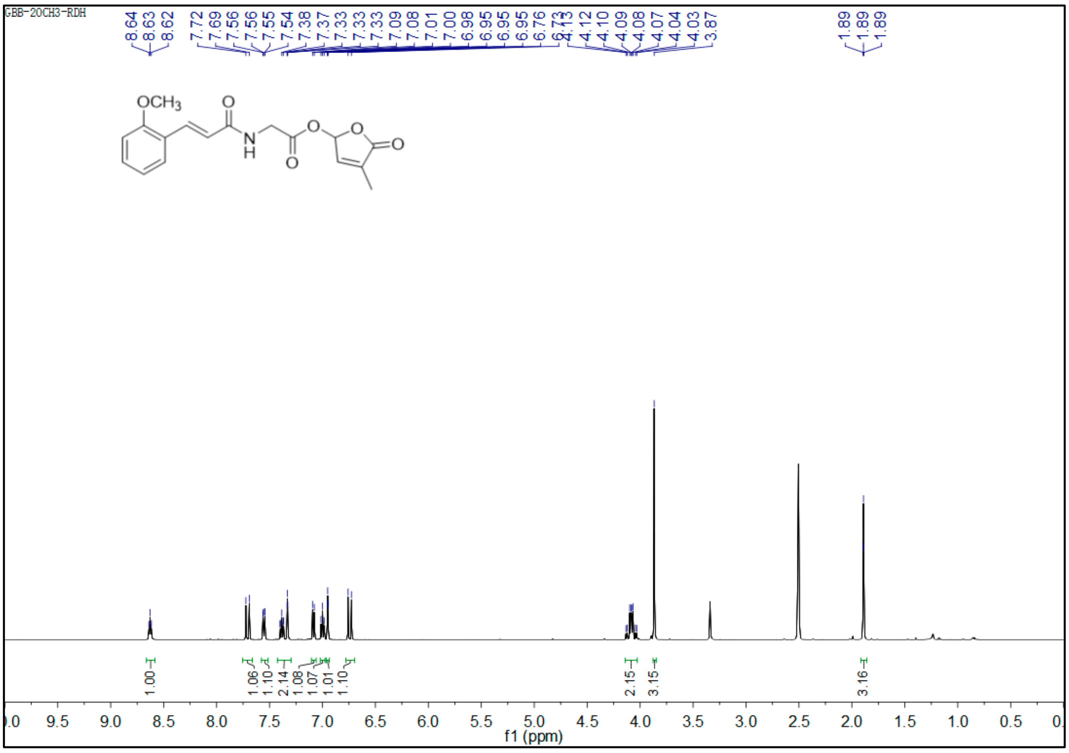

^{1}H NMR spectra of compound **13**

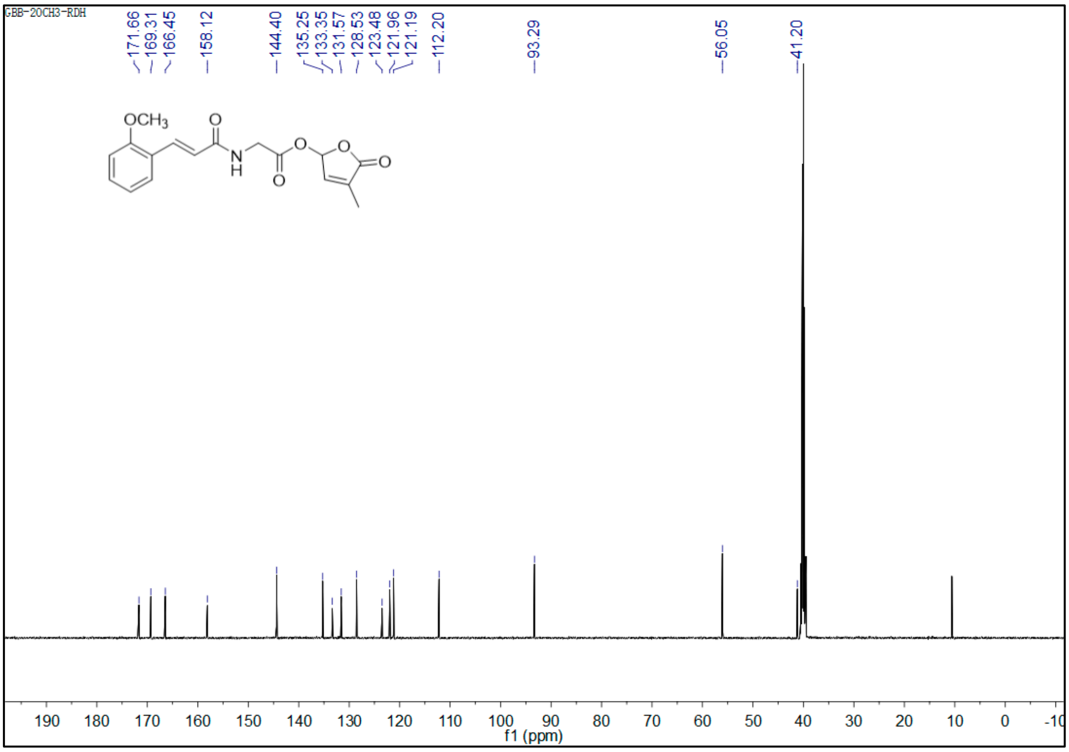

^{13}C NMR spectra of compound **13**

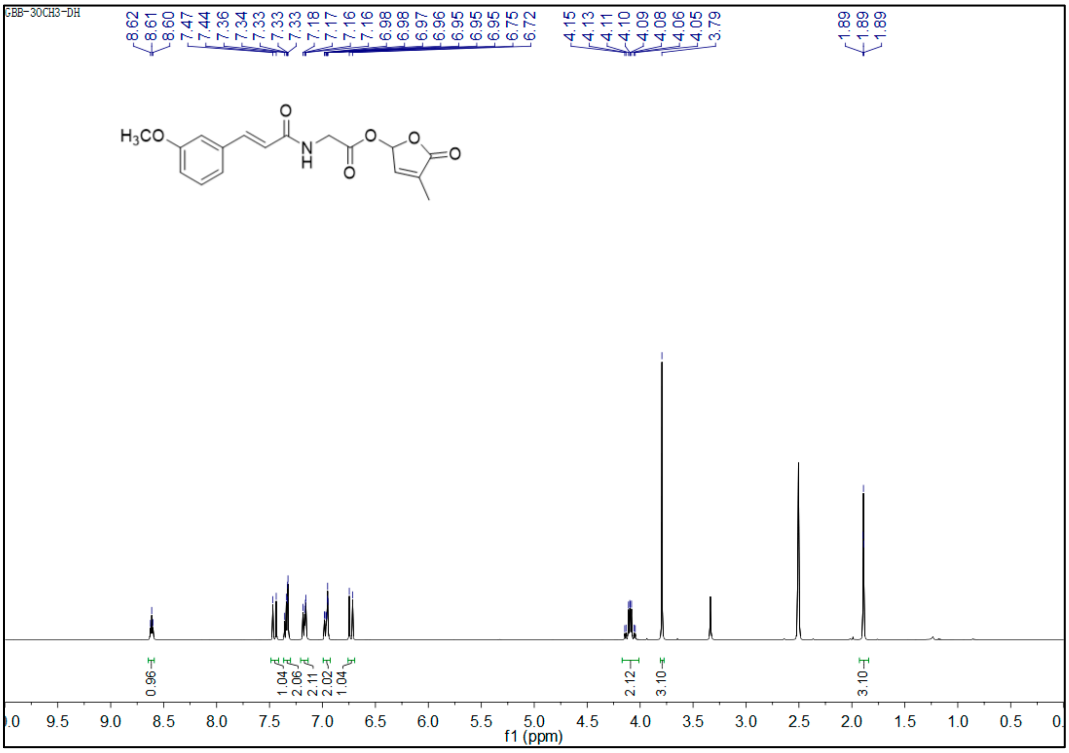

^{1}H NMR spectra of compound **14**

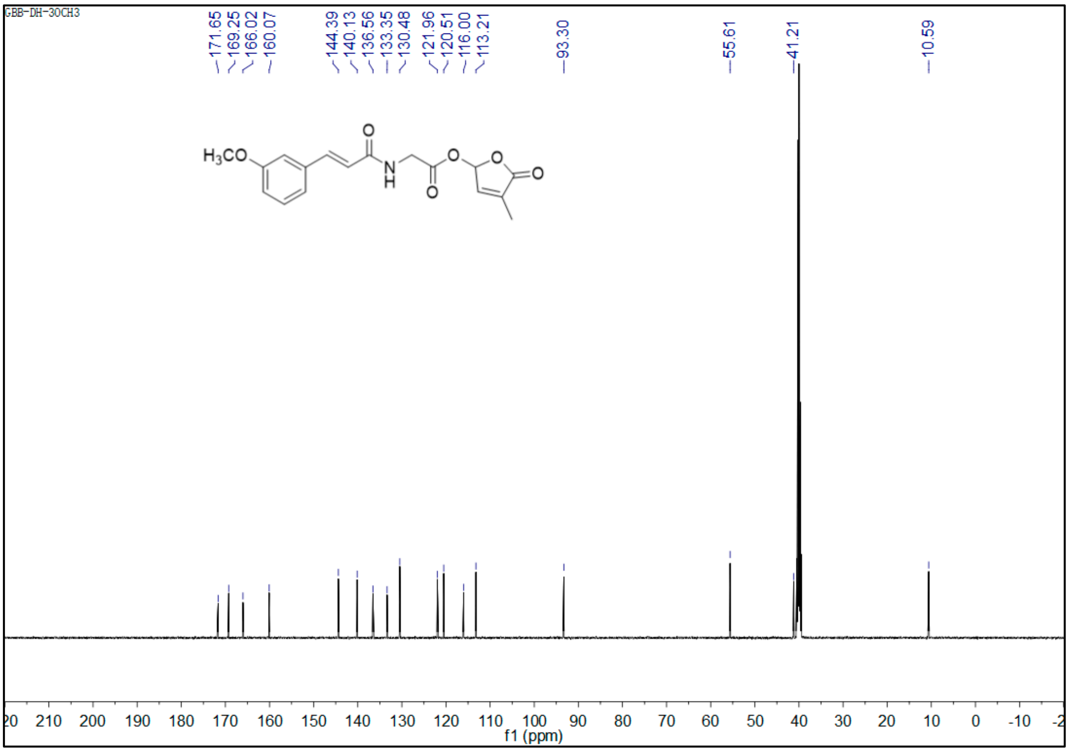

^{13}C NMR spectra of compound **14**

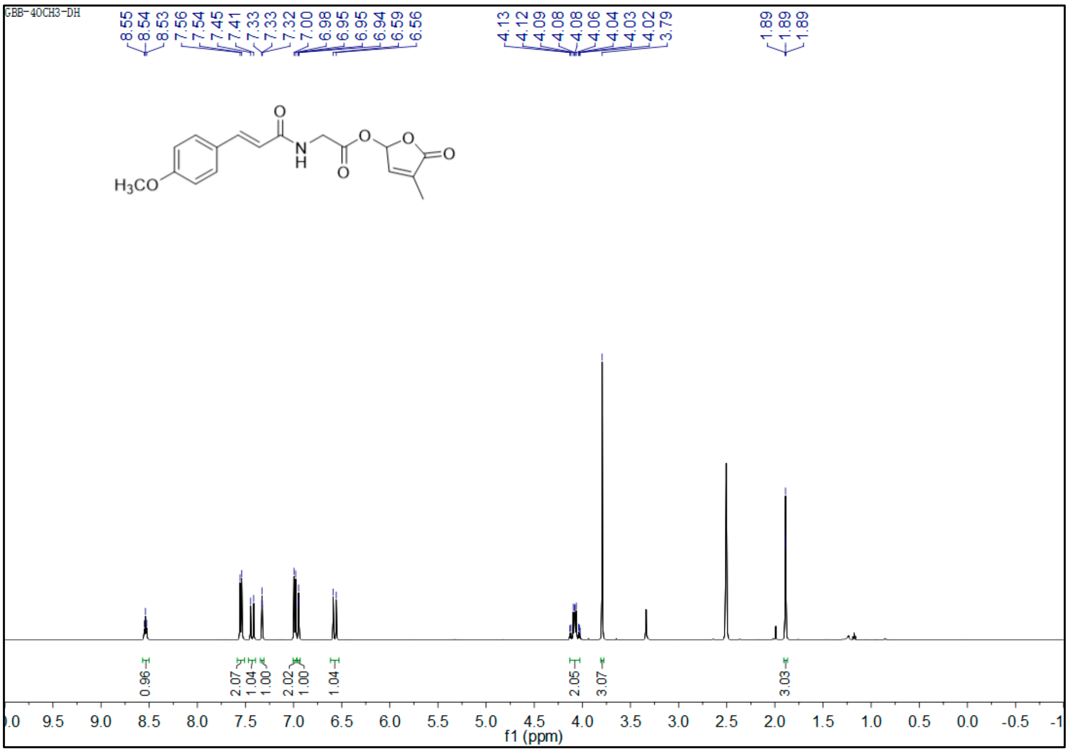

^{1}H NMR spectra of compound **15**

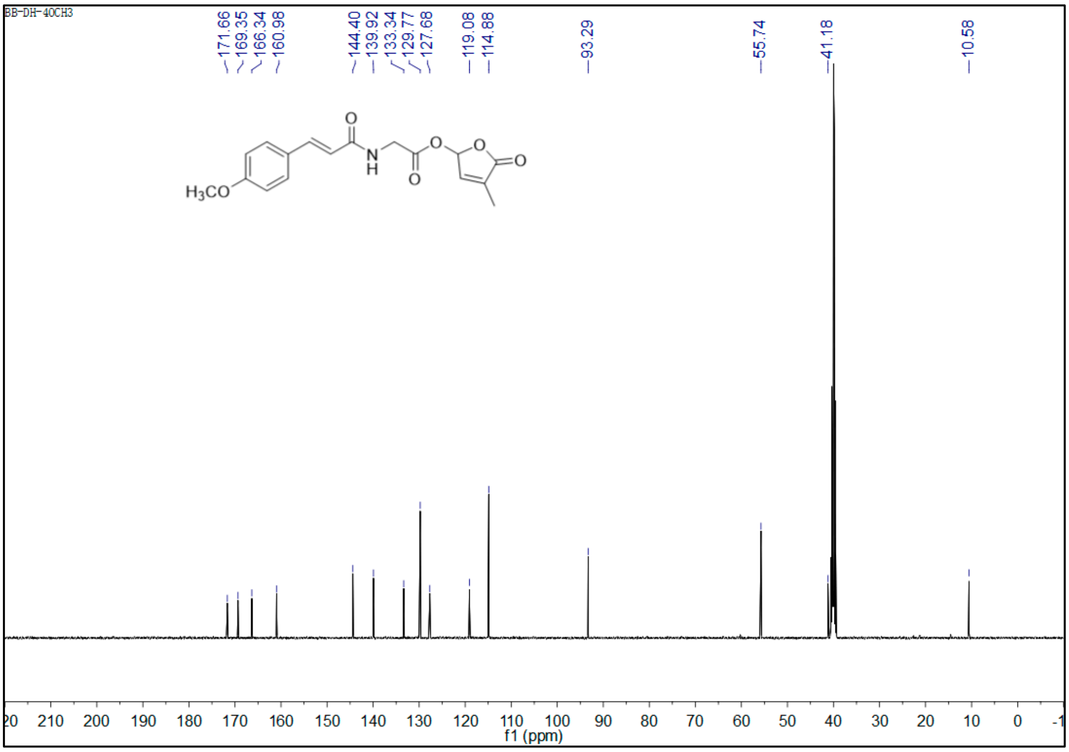

^{13}C NMR spectra of compound **15**

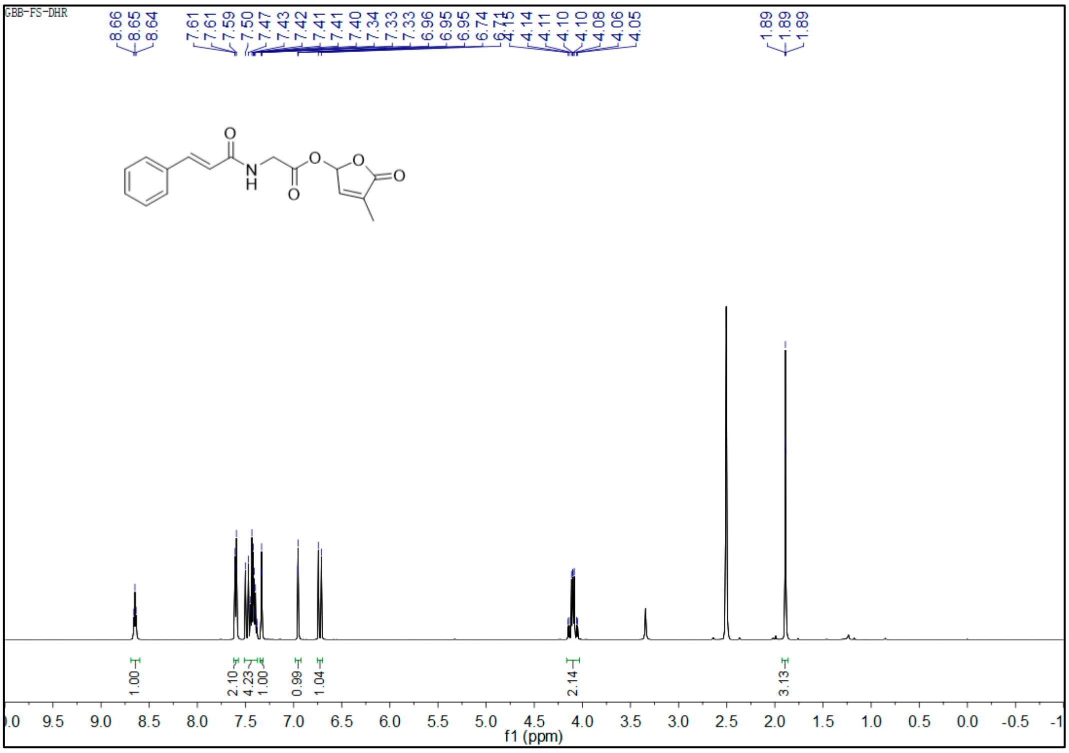

^{1}H NMR spectra of compound **16**

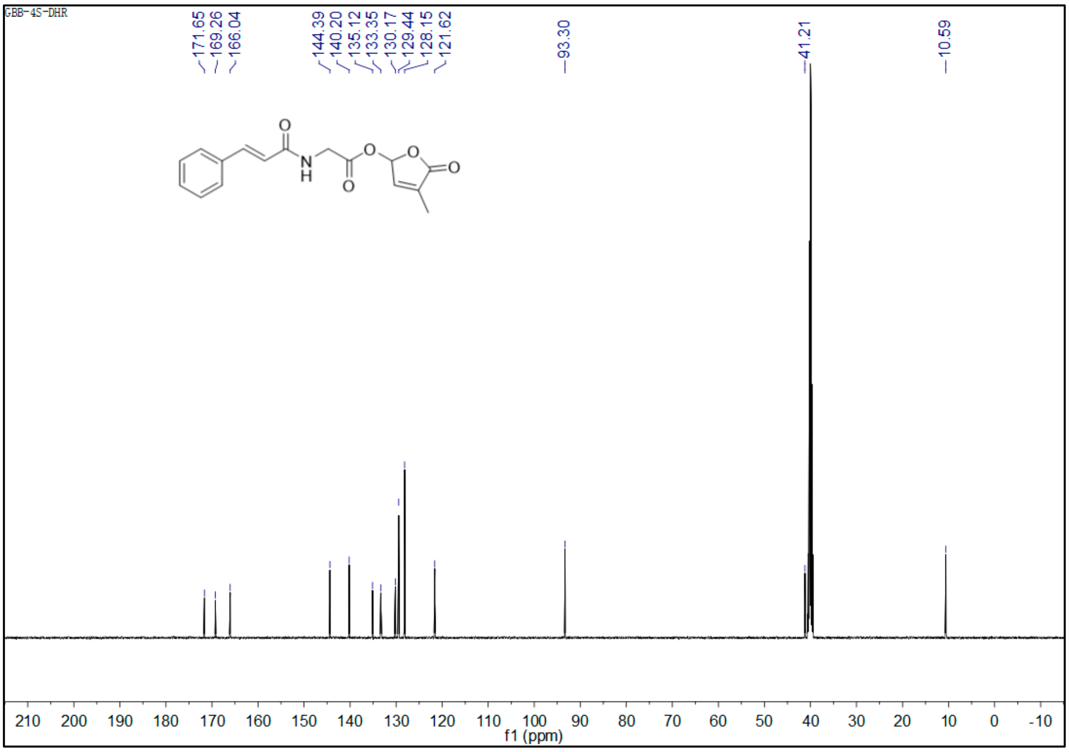

^{13}C NMR spectra of compound **16**

HRMS of compound **1**

Sample No.	Formula (M)	Ion Formula	Measured m/z	Calc m/z	Diff (ppm)
**1**	$C_{16}H_{14}FNO_5$	$C_{16}H_{15}FNO_5$	320.0933	320.0929	1.25

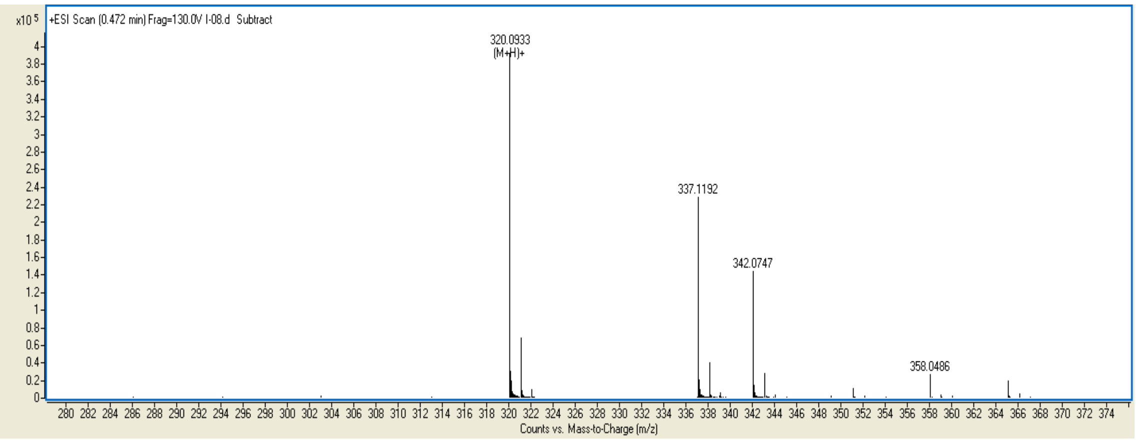

HRMS of compound **2**

Sample No.	Formula (M)	Ion Formula	Measured m/z	Calc m/z	Diff (ppm)
**2**	$C_{16}H_{14}FNO_5$	$C_{16}H_{15}FNO_5$	320.0930	320.0929	0.31

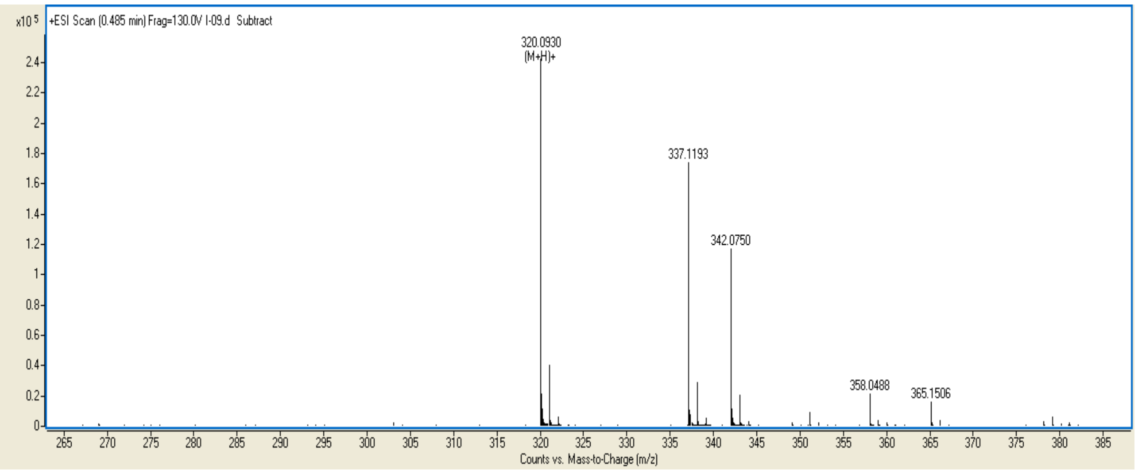

HRMS of compound **3**

Sample No.	Formula (M)	Ion Formula	Measured m/z	Calc m/z	Diff (ppm)
**3**	$C_{16}H_{14}FNO_5$	$C_{16}H_{15}FNO_5$	320.0929	320.0929	0

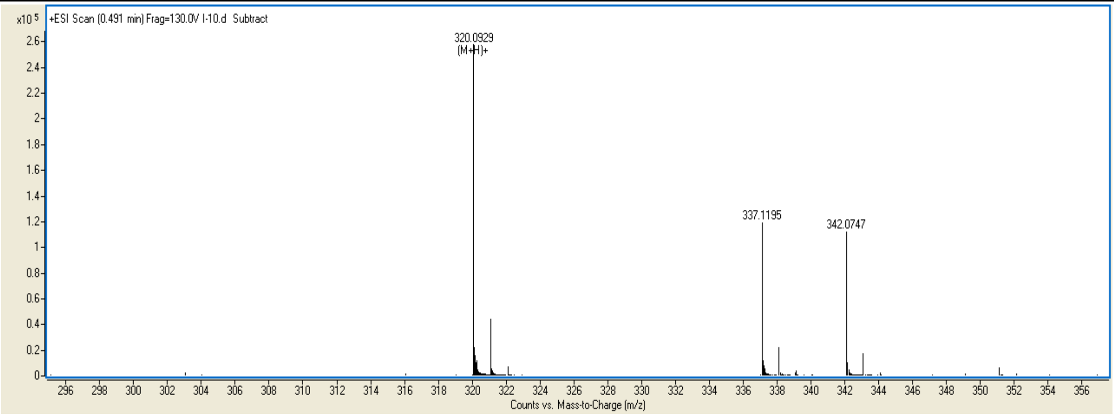

HRMS of compound **4**

Sample No.	Formula (M)	Ion Formula	Measured m/z	Calc m/z	Diff (ppm)
**4**	$C_{16}H_{14}ClNO_5$	$C_{16}H_{15}ClNNaO_5$	358.0453	358.0453	0

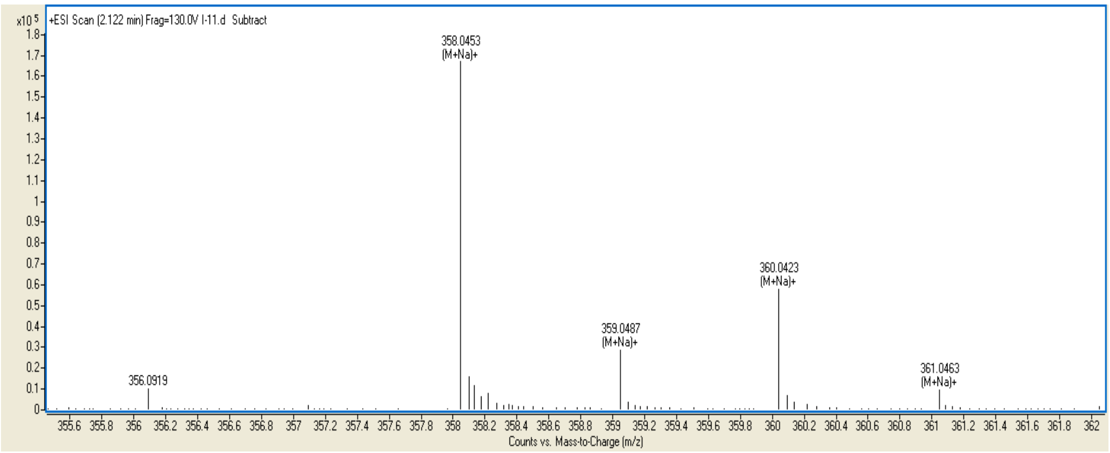

HRMS of compound **5**

Sample No.	Formula (M)	Ion Formula	Measured m/z	Calc m/z	Diff (ppm)
**5**	$C_{16}H_{14}ClNO_5$	$C_{16}H_{15}ClNO_5$	336.0632	336.0633	0.30

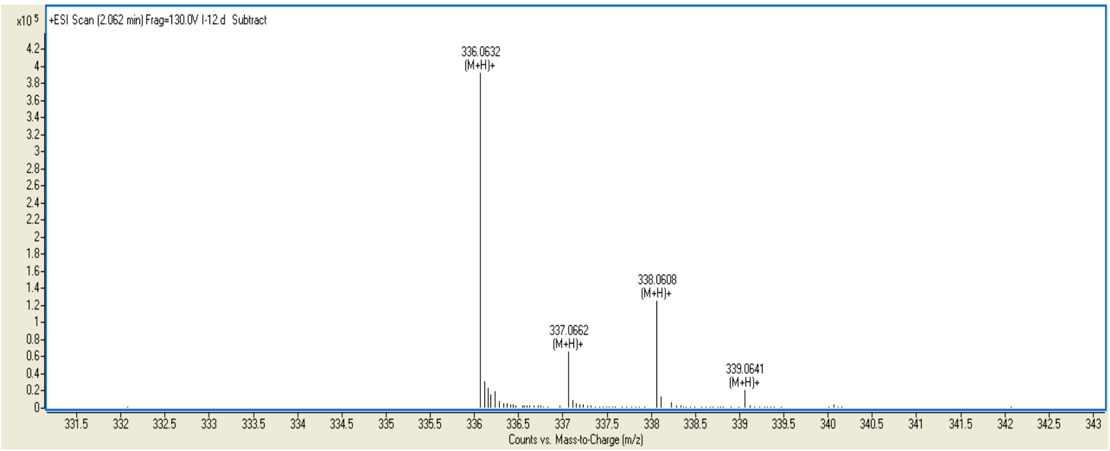

HRMS of compound **6**

Sample No.	Formula (M)	Ion Formula	Measured m/z	Calc m/z	Diff (ppm)
**6**	$C_{16}H_{14}ClNO_5$	$C_{16}H_{15}ClNO_5$	336.0636	336.0633	0.90

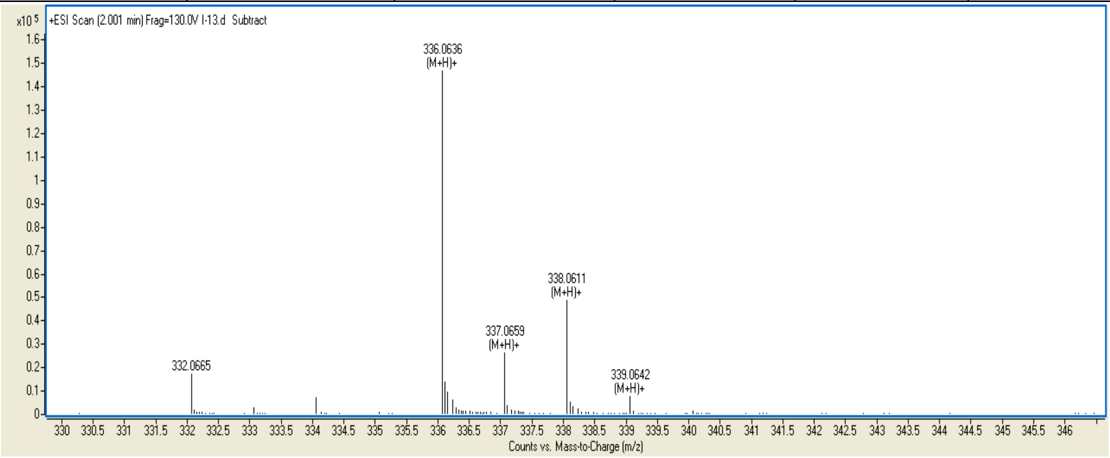

HRMS of compound **7**

Sample No.	Formula (M)	Ion Formula	Measured m/z	Calc m/z	Diff (ppm)
**7**	$C_{16}H_{14}BrNO_5$	$C_{16}H_{14}BrNO_5[M+NH4]^+$	397.0393	397.0394	0.25

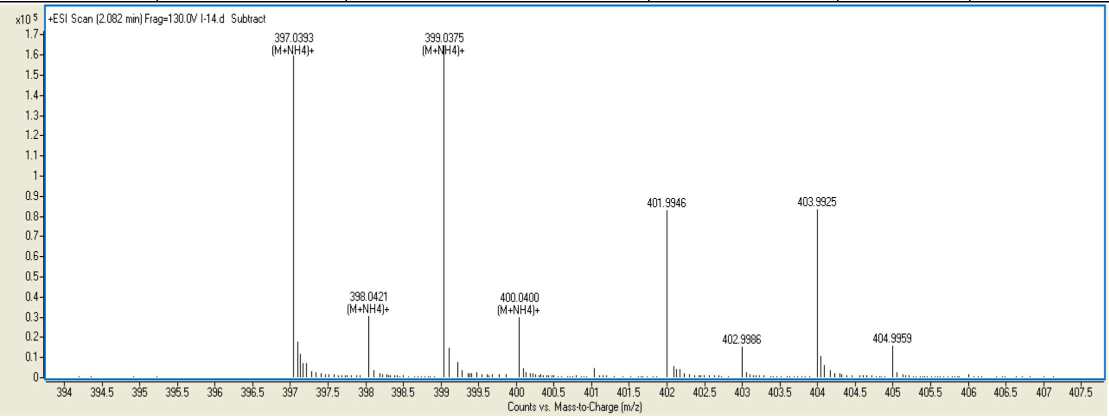

HRMS of compound **8**

Sample No.	Formula (M)	Ion Formula	Measured m/z	Calc m/z	Diff (ppm)
**8**	$C_{16}H_{14}BrNO_5$	$C_{16}H_{15}BrNO_5$	380.0128	380.0128	0

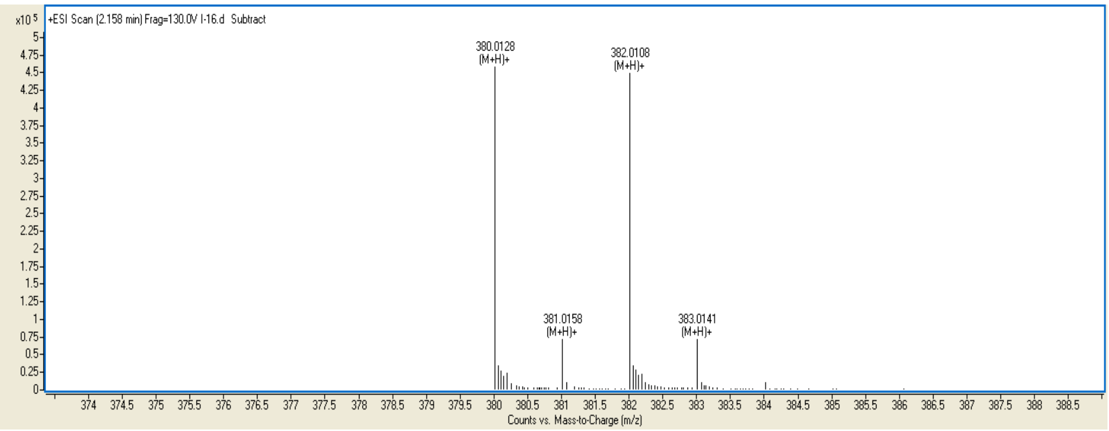

HRMS of compound **9**

Sample No.	Formula (M)	Ion Formula	Measured m/z	Calc m/z	Diff (ppm)
**9**	$C_{16}H_{14}BrNO_5$	$C_{16}H_{15}BrNO_5$	380.0128	380.0128	0

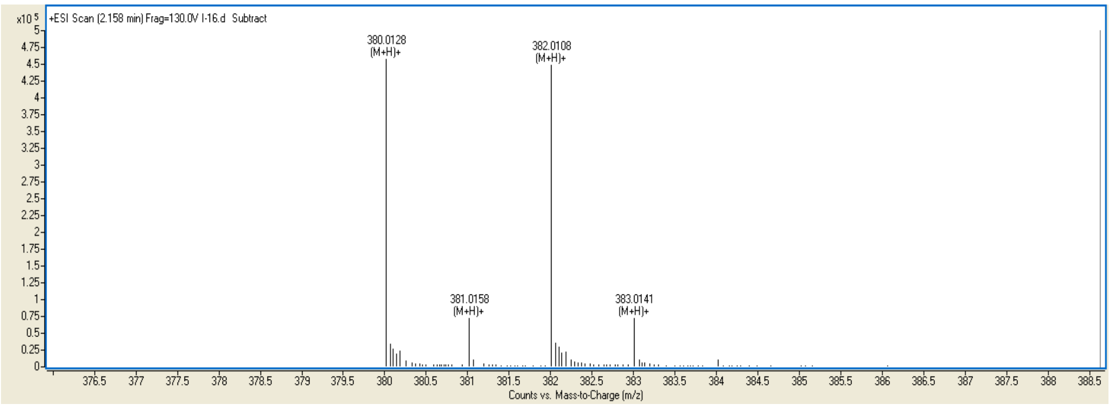

HRMS of compound **10**

Sample No.	Formula (M)	Ion Formula	Measured m/z	Calc m/z	Diff (ppm)
**10**	$C_{17}H_{17}NO_5$	$C_{17}H_{18}NO_5$	316.1182	316.1179	0.95

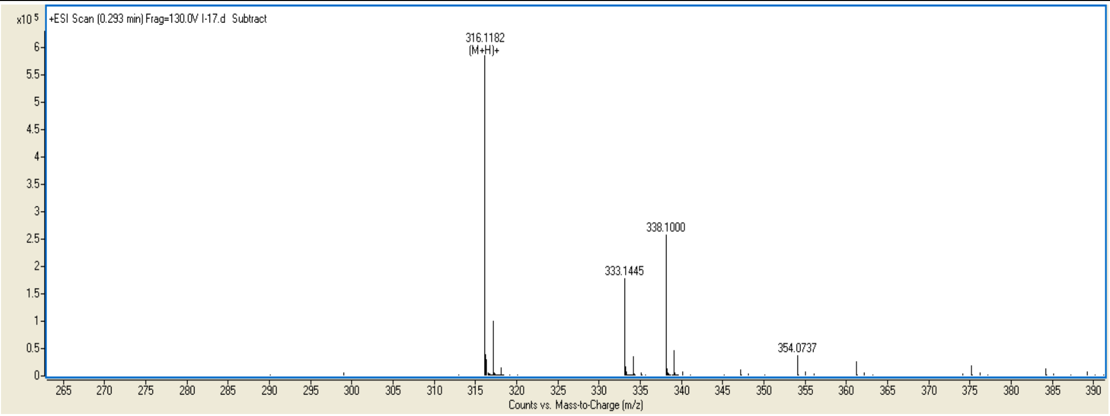

HRMS of compound **11**

Sample No.	Formula (M)	Ion Formula	Measured m/z	Calc m/z	Diff (ppm)
**11**	$C_{17}H_{17}NO_5$	$C_{17}H_{18}NO_5$	316.1179	316.1179	0

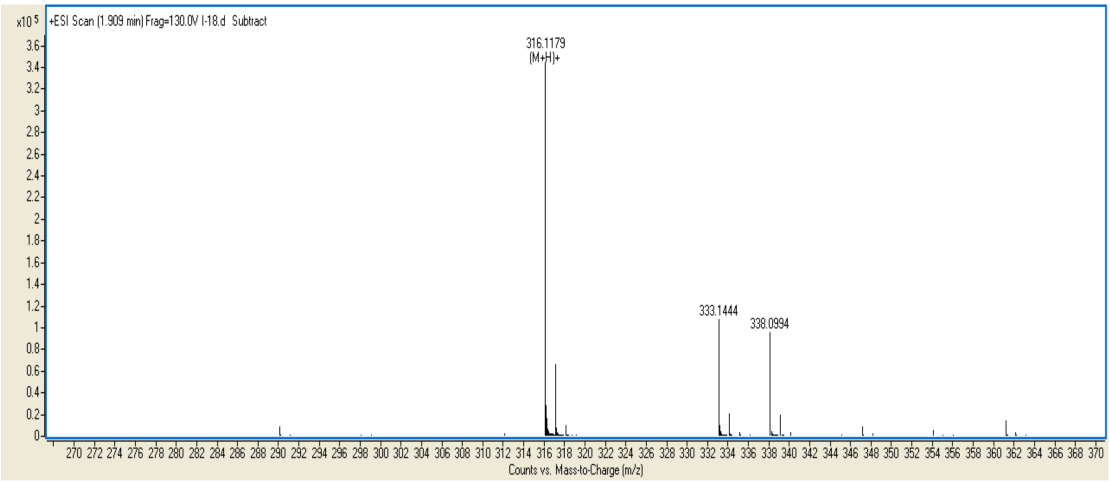

HRMS of compound **12**

Sample No.	Formula (M)	Ion Formula	Measured m/z	Calc m/z	Diff (ppm)
**12**	$C_{17}H_{17}NO_5$	$C_{17}H_{18}NO_5$	316.1179	316.1179	0

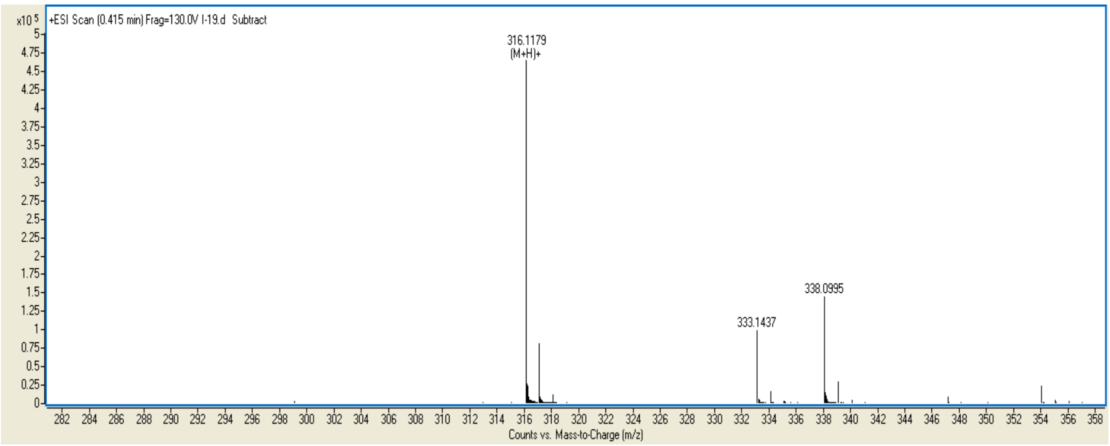

HRMS of compound **13**

Sample No.	Formula (M)	Ion Formula	Measured m/z	Calc m/z	Diff (ppm)
**13**	$C_{17}H_{17}NO_6$	$C_{17}H_{18}NO_6$	332.1128	332.1129	0.3

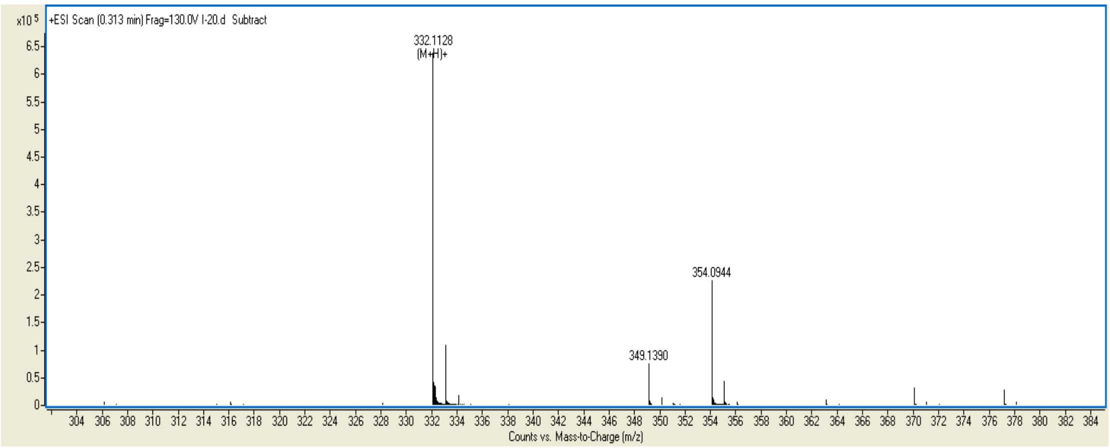

HRMS of compound **14**

Sample No.	Formula (M)	Ion Formula	Measured m/z	Calc m/z	Diff (ppm)
**14**	$C_{17}H_{17}NO_6$	$C_{17}H_{18}NO_6$	332.1129	332.1129	0

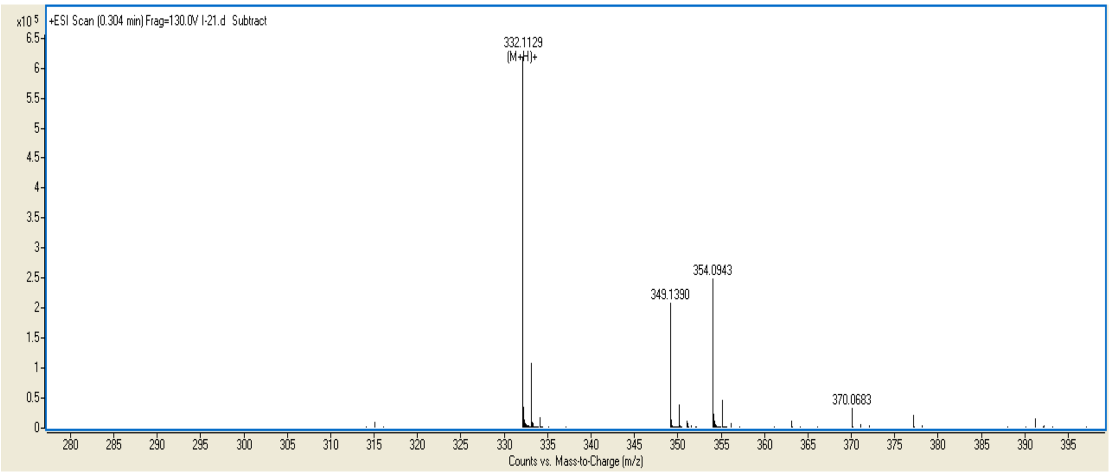

HRMS of compound **15**

Sample No.	Formula (M)	Ion Formula	Measured m/z	Calc m/z	Diff (ppm)
**15**	$C_{17}H_{17}NO_6$	$C_{17}H_{18}NO_6$	332.1128	332.1129	0.3

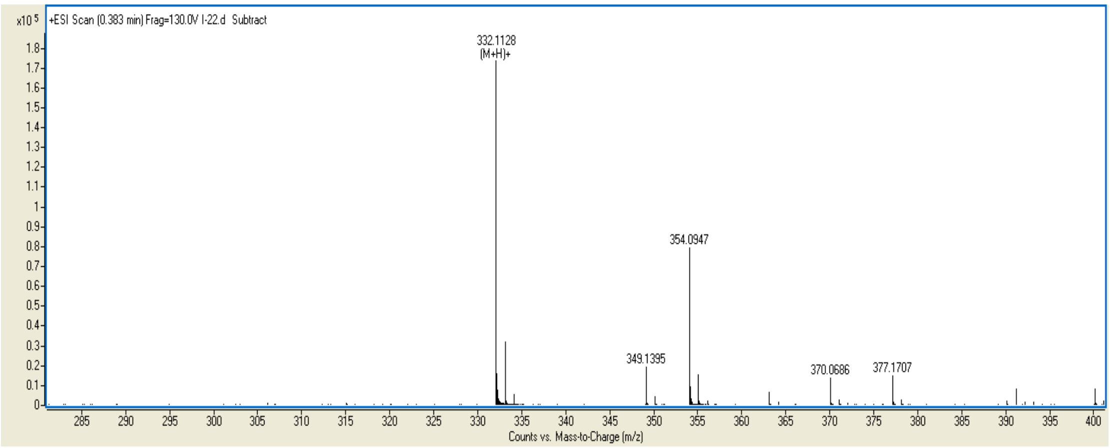

HRMS of compound **16**

Sample No.	Formula (M)	Ion Formula	Measured m/z	Calc m/z	Diff (ppm)
**16**	$C_{16}H_{15}NO_5$	$C_{16}H_{16}NO_5$	302.1023	302.1023	0

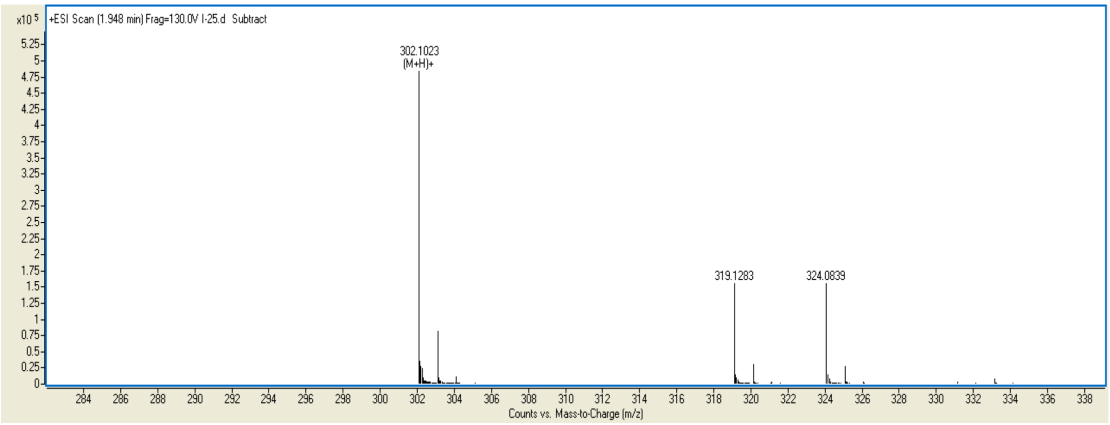

Supplement: Supplementary file 1 [file ijms-24-09967-s001.zip › ijms-2393188-supplementary.pdf]
